# Probing Conformational Stability and Dynamics of Erythroid and Nonerythroid Spectrin: Effects of Urea and Guanidine Hydrochloride

**DOI:** 10.1371/journal.pone.0116991

**Published:** 2015-01-24

**Authors:** Malay Patra, Chaitali Mukhopadhyay, Abhijit Chakrabarti

**Affiliations:** 1 Chemistry Department, University of Calcutta, Kolkata, West Bengal, India; 2 Crystallography & Molecular Biology Division, Saha Institute of Nuclear Physics, Kolkata, West Bengal, India; CNR, ITALY

## Abstract

We have studied the conformational stability of the two homologous membrane skeletal proteins, the erythroid and non-erythroid spectrins, in their dimeric and tetrameric forms respectively during unfolding in the presence of urea and guanidine hydrochloride (GuHCl). Fluorescence and circular dichroism (CD) spectroscopy have been used to study the changes of intrinsic tryptophan fluorescence, anisotropy, far UV-CD and extrinsic fluorescence of bound 1-anilinonapthalene-8-sulfonic acid (ANS). Chemical unfolding of both proteins were reversible and could be described as a two state transition. The folded erythroid spectrin and non-erythroid spectrin were directly converted to unfolded monomer without formation of any intermediate. Fluorescence quenching, anisotropy, ANS binding and dynamic light scattering data suggest that in presence of low concentrations of the denaturants (up-to 1M) hydrogen bonding network and van der Waals interaction play a role inducing changes in quaternary as well as tertiary structures without complete dissociation of the subunits. This is the first report of two large worm like, multi-domain proteins obeying twofold rule which is commonly found in small globular proteins. The free energy of stabilization (ΔG_u_^H^_2_^0^) for the dimeric spectrin has been 20 kcal/mol lesser than the tetrameric from.

## Introduction

Understanding of the stability of a protein under different chemical conditions is crucial for protein chemists and also biologists. In the past few years the scientists have been trying to find the mechanism through which proteins maintain their biologically active three dimensional structures both by theoretical and experimental studies [1, 2]. The folded state of a protein in solution is favored through combinations of electrostatic; van der Waals, hydrogen bonding and hydrophobic interactions among amino acid residues to overcome the loss of entropy during folding [3]. Proteins are known to assume different conformations during denaturation and the denatured states are equally important as that of the native state in determining stability of proteins [4, 5]. Urea and guanidine hychloride are commonly used as strong denaturants of proteins and difference in stability of protein in the native and unfolded states are studied in the presence and absence of these chaotropic agents although their mechanism of action is still obscure [6, 7]. It is still an open question whether they exert their effect by direct binding to the protein or they act indirectly by altering the 3D network of water [8, 9]. Multi domain proteins are encoded by two-thirds of the eukaryotic genome [10, 11]. The conformational stability of these mulitidomain proteins can be monitored through equilibrium unfolding studies of individual structural domains using urea and GuHCl [12]. But recent studies indicate that in a multi-domain protein, an individual domain could fold in a co-operative manner, i.e. the folding of one domain is influenced by the other, whereas in others, the individual domain might fold independently [13, 14, 15, 16].

Spectrin domains fold in a co-operative fashion, form a dense two dimensional filamentous network that provides support to the lipid bilayer and maintains erythrocyte shape. It is an amphiphilic, elongated heterodimer with two (α and β) subunits maintaining integrity and architecture of the red blood cell. Several blood diseases are associated with erythrocyte deformation and defects in spectrin e. g. hereditary spherocytosis; a type of hemolytic anemia involves mutation in β-spectrin [17, 18, 19]. Spectrin has also been implicated to play important roles in the maintenance of the erythrocyte membrane asymmetry and exhibits chaperon like activity [20, 21]. The two subunits of spectrin are homologous with about 30% identity and are aligned in an antiparallel side to side orientation to give a flexible rod-shaped molecule. These heterodimers, associate head-to-head to form tetramer or higher order oligomers. The primary structure of spectrin is comprised of homologous units (spectrin repeats) of 106 amino acids in length. The repeats fold into coiled coil of three anti parallel left handed helices. These triple helical units are connected through a helical linker. The intact spectrin molecule is made up of about 90% of these 3-helix repeat (Usually 20 α- and 17 β-chains). The X-ray structure of a single repeat motif shows that the 3-helix bundles are stabilized through hydrophobic interaction between the interior hydrophobic surface of the amphiphilic helix and electrostatic interactions between the charged surfaces. In a tandem array of these spectrin repeat domains the last helix of the first domain is seen to form a continuous helix with the first helix of second repeat domains. These domains are stabilized both by their neighbors and the co-operativity of these spectrin domains has been attributed to the presence of linking helical propensity [22, 23]. The 16th and 17th repeat domains are the most studied structural domain of both spectrins (erythroid and no-erythroid, brain spectrin) since these two or three repeat domains of spectrin governs the overall flexibility of this ubiquitous membrane skeletal protein. Crystal structures of R16 and 17 domains show a long connecting helix between the two domains consisting of helix C from R16 and helix A from R17 [24]. In a number of studies Macdonald and co-workers have demonstrated that in solution individual domain can fold cooperatively and reversibly to stable structure. From thermodynamic studies they have suggested that the helical propensity of linker between the domain pairs correlates well with the relative stability of a single domain [25]. It has been shown through kinetic and equilibrium studies that the two tandem repeats act as a classical two fold system and stability of the each domain in the two domain-pair contributes to the overall stability of the protein [26, 27]. This zigzag arrangement of helices with amino and carboxy terminal residues at opposite hand in a 3-helix bundle are thought to be linked in tandem in spectrin, producing a large dimeric molecule approximately 100 nm in length as seen under electron microscope. These structural features allow spectrin to take part in many physiological events through protein-protein interactions and the ability of spectrin to expand and contract has been attributed to its modular structure. Conformational studies on spectrin indicate that the protein contains rigid and globular domain and also a portion of flexible, segmentally mobile conformation which is highly hydrophobic in composition.

The other form of non-erythroid spectrin, found in neuronal tissue, is very similar in structure and has less defined function. The N-terminal region of α-subunit and C-terminal region of β-spectrin of nonerythroid spectrin exhibit about 60% and 70% sequence similarity with those of erythroid spectrin [28, 29]. However, the two spectrin isoforms are quite different in terms of their structure and stability. Erythroid spectrin is easily capable of head to head association to form the tetramer and higher order oligomers under physiological condition through sequential addition of heterodimers. Equilibrium sedimentation, electron microscopy and nondenaturing electrophoresis experiments, on the other hand, reveal that nonerythroid spectrin isofrom predominantly exits as a tetramer [30, 31]. Crystallographic structural data reveals that the tetramerization site or the self-associating domain of nonerythroid α-spectrin is different from that of erythroid spectrin. This supports that the heterodimer of nonerythroid spectrin forms tetramer about 15 times stronger than erythroid spectrin [32]. Nonerythroid spectrin is found to be more rigid and thermally stable than the erythroid spectrin and interacts more strongly with anionic lipid membrane than erythroid spectrin [33, 34].

To consider stability of such multidomain proteins, most of the earlier studies were carried out with one or two repeat motifs of 15^th^, 16^th^ and 17^th^ domain of both forms of spectrin [13, 14, 25, 26, 35, 36, 37]. A single motif composed of 106 amino acid residues was found to be a compact and proteolytically resistant unit. Similar units composed of two or three motifs in tandem with the linker region were shown to be substantially more stable through such interactive stabilization [38]. We have realized the importance to examine the stability of both forms of spectrin in their intact dimeric and tetrameric form in addition to all previous work done with the individual repeat motifs [13, 14, 25, 26, 35, 36, 37, 38]. The unfolding transition midpoint of intact brain spectrin and their subunits have been found to be elevated by 10°C more than that of the erythroid spectrin using far UV CD measurements [39].

Spectrin has large number of tryptophan residues spread over the entire length of the dimer. It is noteworthy that typically 106 amino acid long repeat units in spectrin have tryptophan’s strongly conserved at 45^th^ position in 20 repeats out of a total 22 segments in α spectrin. In all 17 repeats of β-spectrin, Trps are present in 45^th^ position and in some repeats in 11^th^ position. Careful examination shows that there are 22 segments in α subunit and 17 segments in β-subunits of a spectrin dimer [40, 41]. Segments 1–9 and 12–19 are homologous and are therefore considered as repeats. Segments 11, 20, 21 and approximately N-terminus 50 amino acid residues from segments 22, although less conserved, are related to other highly conserved repeats. Of these most homologues segments, 11^th^ are 106 amino acids long. There are all together 42 Trps in the 20 repeats of α-spectrin and 35 Trps in 17 repeats of the β-spectrin in erythriod spectrin dimer. The Trps in the repeat motifs represent more than 90% of the total Trps in the spectrin dimer. In addition there are 5 tryptophans in the acting binding and 2 tryptophans at the carboxy terminus of β-spectrin. Nonerythroid spectrin contain conserved tryptophan at the 45^th^ and 53^rd^ positions in altogether 21 repeat motifs of α-spectrin and at 45^th^ positions in 17 repeats of β-spectrin respectively [42]. Some of these conserved tryptophan residues have been shown to promote the folding of spectrin domains and contribute to their thermodynamic stability [43, 44]. Such localization of the Trps in the same position in each domain makes them convenient intrinsic fluorescence reporter groups for the study of conformational stability, shown in our earlier work [45, 46, 47]. In the present study, we focus on studying the conformational stability and structural dynamics of the dimeric, tetrameric erythroid and tetrameric nonerythroid spectrins using spectroscopic techniques. Steady state and time-resolved fluorescence, steady state anisotropy, acrylamide quenching and circular dichroism spectroscopy have been used to monitor the tertiary and secondary structural changes of the two proteins in presence and absence of urea and GuHCl. Analysis of unfolding data along with available structural information indicates that folded forms of spectrin are more susceptible to GuHCl compared to urea indicating charged residues on the surface of the proteins to play fundamental role for thermal tolerance of both forms of spectrin. Our data suggest that unfolding of both tetrameric and dimeric proteins follow two fold models like mesophilic monomeric globular protein. Thermodynamic data showed higher stability of the tetrameric both erythroid and nonerythroid spectrin than the dimeric erythroid spectrin, suggesting the potential of the hinge region to provide additional stability to the nonerythroid tetrameric spectrin also shown previously using recombinant erythorid tetramers [38].

## Materials and Methods

Ovine blood and brain tissue samples were obtained from local slaughterhouse/ meat shop, Salt Lake Slaughter House in the CK Market. Blood samples were collected in EDTA and brain tissues from freshly slaughtered animals and were used for the purification of both forms of spectrin, elaborated in our earlier work [47]. Tris, KCl, phenyl methylsulfonyl fluoride (PMSF), dithiothreitol (DTT), EDTA, Imidazole, MgCl_2,_ NaCl, N-acetyl-tryptophanamide (NATA), urea, GuHCl and acrylamide were obtained from Sigma-Aldrich Chemical Co. St. Louis, Mo, USA. Potassium Iodide was purchased from E-Merck, Mumbai, India. ANS was obtained from Molecular Probe. All other chemicals used were of the highest quality available. De-ionized water from Milli Q (Millipore Corporation, USA) was used for the preparation of the buffer and all other solutions.

### Isolation and purification of erythroid and non-erythroid spectrin

Clean white ghosts from ovine blood were prepared by hypotonic lysis in 5mM phosphate; 1mM EDTA, containing 20μg/ml phenylmethyl sulfonyl fluoride at pH (lysis buffer) 8.0. Spectrin dimers were purified from ovine erythrocytes following published procedure elaborated earlier [47, 48]. After washing the membrane thoroughly lysis buffer, they were suspended in 20 volume of spectrin removal buffer (0.2mM of sodium phosphate, 0.1mM EDTA 0.2 mM DTT 20 μg/ml PMSF, pH 8.0 ) at 37°C for 30 minutes for the purification of dimeric spectrin. The tetrameric spectrin were purified by following the same method by resuspending the membranes in 20 volumes of spectrin removal buffer at 4°C and were dialyzed against the same buffer for purification of spectrin tetramer. The crude spectrin was collected in supernatant after centrifugation. Both dimeric and tertrameric spectrin were then purified after concentration by 30% ammonium sulphate precipitation followed by chromatography on Sepharose CL-4B. Spectrin was stored in a buffer containing 5 mM Phosphate 20 mM KCl, 1mM EDTA pH 8.0 containing 0.2mM DTT. Concentrations of dimeric, spectrin were determined spectrophotometrically using an absorbance 10.7 at 280 nm for 1% spectrin solution and the concentration of dimeric and tetrameric spectrin was determined by Bradford method.

Nonerythroid spectrin in its tetrameric from was purified from ovine brain, following published procedure [48, 49]. Freshly homogenized brain was further homogenized in 10 mM Tris at pH 8.0 containing 5mM MgCl_2_ 1mM EGTA 0.2 mM DTT and 0.2mM PMSF. The salt concentration of the solution was maintained at 0.6M from stock NaCl solution and was stirred for 1hr at 4°C. The crude nonerythroid spectrin obtained after centrifugation at 12000g at 4°C. Brain spectrin after concentrating with 50% ammonium sulphate precipitation was further purified following chromatography on Sepharose CL-4B. Purity of the preparation was checked by 7.5% SDS-PAGE under reducing condition which showed characteristic bands of spectrin subunits after staining with coomassie blue. Concentration of nonerythroid spectrin was determined by Bradford method [50]. Before all spectroscopic experiments, spectrin was dialyzed extensively against 10 mM Tris 20mM KCl at pH 8.0 to remove DTT and EDTA.

### Steady state fluorescence measurements

For all steady state fluorescence measurements 0.2 mg/ml of protein (erythroid / nonerythroid spectrin) solution was used in 10 mM Tris 20 mM KCl buffer, pH 8.0, having different concentration of urea or GuHCl. For denaturation studies the protein solution having different concentration urea (from 0–8M) or GuHCl (from 0–6M) were incubated for 1 hour at 25°C.

Steady state fluorescence measurements were carried out in Cary Eclipse fluorescence spectrophotometer equipped with thermostated cell holders, keeping constant temperature using circulating water bath. Fluorescence measurements were made by excitation both at 280 nm and at 295 nm wave length selectively for the tryptophan residues. Excitation and emission slit with a nominal band pass 5 nm were used for all measurements. Background intensities without spectrin were subtracted from each to cancel out any contribution due to solvent Raman peak and other scattering artifacts. Both the changes in intensity and the shift of emission maxima were recorded to monitor the conformational changes of protein.

Steady state Fluorescence anisotropy (r) measurements were performed using Cary Eclipsed Polarization accessory. Anisotropy value was calculated from the equation [51].

$${r=I}_{\mathrm{VV}}{-\mathrm{GI}}_{\mathrm{VH}}{/I}_{\mathrm{VV}}{+2\mathrm{GI}}_{\mathrm{VH}}$$ (1)

Where I_VV_ and I_VH_ are the measured fluorescence intensity with excitation polarizer vertically and emission polarizer vertically and horizontally oriented respectively. The factor G is defined as

$${G=I}_{\mathrm{HV}}{/I}_{\mathrm{HH}}$$ (2)

I_HV_ is the fluorescence intensity with excitation polarizer horizontally and emission polarizer vertically oriented. I_HH_ is the fluorescence intensity where both polarizers horizontally oriented. All experiment was done with multiple sets of sample and average 5 independent measurements of anisotropy are summarized.

### Time resolved fluorescence measurements

Fluorescence lifetime measurements were made by time correlated single photon counting on Fluromax-3 spectrophotometer using Nano LED as a light source. To optimize signal to noise ratio 5000 counts were collected in the peak channel. All the experiments were performed in a buffer containing 10mM Tris and 20mM KCl, pH 8.0 using excitation and emission slits with a nominal band pass of 4nm or less. The excitation and emission wavelengths were fixed at 295nm and 340nm respectively for monitoring the lifetime tryptophan for erythroid and nonerythroid spectrin, elaborated earlier [48].

### Quenching of tryptophan fluorescence

Quenching of tryptophan fluorescence were carried out by exciting at 295 nm and record emission intensities at 340 nm after serial addition of small aliquots of stock solution of the quenchers (2M), acrylamide and iodide [52, 53, 54]. Both forms of spectrin (0.1mg/ml) were incubated with different concentrations of the denaturant before the quenching experiments were carried out. The quenching data were analyzed using Stern-Volmer equation

$$F_{0}{/F}_{\mathrm{corr}}{=1+K}_{\mathrm{sv}}\left[Q\right]$$ (3)

Where F_0_ is the fluorescence intensity in absence of the quencher, F_corr_ is the same at quencher concentration of Q and K_SV_ is the Stern-Volmer quenching constant, the bimolecular quenching constant which can be interpreted in different ways depending on the assumed mechanism of quenching. An estimate of the accessibility of the Trp residues by the quencher was obtained from the following equation

$$F_{0}/\left(F_{0}{-F}_{\mathrm{corr}}\right)=1/\left(K_{\mathrm{SV}}f_{e}\left[Q\right]\right){+1/f}_{\text{e}}.$$ (4)

Where f_e_ is the total number of Trp residues in the protein those are accessible to the quenchers. Quenching experiment was also carried out by time resolved fluorescence measurement when τ_0_/τ was plotted against the quencher concentration, τ_0_ and τ being the mean lifetime values in the absence and presence of the quencher, respectively.

### Circular Dichroism (CD) measurements:

CD spectra were recorded on a Biologic CD spectrometer using a quartz cell of 1mm (0.1 mm) pathlength at 25°C. For recording spectra in the far-UV region, scanning was made in the range between 190 nm and 250 nm at a rate of 3nm / min. Each spectrum shown, is average of five continuous scans corrected by subtraction of appropriate blank without spectrin. Both forms of spectrin (0.1mg/ml) in a buffer of 10 mM Tris, 20 mM KCl, pH 8.0 was incubated for 1 hour with different concentrations of the denaturants (urea/GuHCl) before the spectra were recorded. The spectra were subjected to moderate degree of noise-signal reduction analysis by smoothening to make sure that the overall spectrum remains unaltered. Intensities were expressed as mean residue ellipticity, the molar ellipticity per mean residue [θ] × (deg cm^2^ dmol^−1^) obtained from the relation

$${\left[\theta \right]}_{222}={\left[\theta \right]}_{\mathrm{obs}}\left(\mathrm{mrw}\right)∕10.c.l$$ (5)

Where [θ]_obs_ is the observed ellipticity in degree, mrw is the mean residue molecular weight of the protein (mrw of 115.2 for erythroid and 115.5 for brain spectrin) [33], c is the protein concentration (g/ml) and l is the optical pathlength of the cell in cm.

### Fluorescence of Spectrin-bound ANS

The extent of exposure of surface hydrophobicity and polarity of the hydrophobic binding site in the protein was measure by its ability to bind ANS. The protein concentration was kept at 0.1mg/ml for the all binding experiments. A stock solution of ANS is prepared in DMF and the concentration was determined using molar absorbance of 7800 at 372 nm [21]. For binding studies, 100-fold molar excess of ANS and the proteins were incubated for 30 minutes at 25°C in dark, in presence and absence of the denaturants, urea and GuHCl. ANS fluorescence was measured upon excitation at 380 nm and the emissions were recorded between 400nm to 600nm.

### Dynamic light scattering measurements

Dynamic light scattering (DLS) experiments are usually done for globular protein to determine the size and shape of the protein from hydrodynamic radius. Budzynaski and coworkers first performed DLS measurements with spectrin at scattering angle from 0° to 130°C [55, 56]. We have also performed DLS measurements to study the effects of the denaturants on overall conformation of both the spectrins. We used bovine serum albumin as positive control. The measurements were performed on a DLS spectrometer of Nano Series from Malvern instruments, equipped with thermostat chamber. The protein concentrations were again maintained 0.1 mg/ml during DLS experiments in presence and absence of urea/GuHCl in a buffer of 10 mM Tris-HCl at pH 8.0. Before carrying out the DLS experiments all solutions were passed through 200 nm membrane filter (Millipore). The analysis of light scattering from diffusing particles enables to determine intensity autocorrelation function <G (t)>, related to normalized electric field autocorrelation [g (t)] through Siegert relation. The mutual diffusion coefficients are calculated from electric field autocorrelation using Laplace transform program (CONTIN) through distribution relaxation rate (Λ) and measurement vector (K) as

$$\mathrm{Dm}=\Gamma /K^{2}$$ (6)

K=4Πµ/λSin(θ/2) (7)

Where μ is the refractive index of the medium (1.33 for water), θ is the angle of scattering, fixed at 173° for all the measurements. From the measurements of the diffusion coefficient, one obtains the apparent Stokes hydrodynamic radius of a molecule using the following relation -

$$d_{H}=k_{B}T/3\pi \eta D$$ (8)

where k_B_ is Boltzmann constant, T is absolute temperature, η is viscosity coefficient of the solvent and D is the translational diffusion coefficient. In a typical histogram showing the size distribution, the X axis indicates size in nanometers and the Y axis shows the relative intensity of scattered light or the relative number of particles.

### Analysis of equilibrium denaturation data

Unfolding of dimeric spectrin was described as a two state transition with the native protein directly forming unfolded monomers [57, 58].

$$\left(N_{2}↔2U\right)$$ (9)

The equilibrium constant of the unfolding reaction, K_u_ and the free energy of unfolding, ΔG_u_ are defined as

$$\mathrm{Dimer}:K_{u=}{\left[U\right]}^{2}/[N_{2}]=2\;P_{t}f_{u}{}^{2}/(1-f_{u})$$ (10)

$$\Delta G_{u}=-RT\;\ln\;K_{u}$$ (11)

Unfolding of tetrameric spectrin was also described as two state transition with native proteins directly converted to denatured monomers.

$$\left(N_{4}↔4U\right)$$ (12)

The equilibrium constant of the unfolding reaction, K_u_ and the free energy of unfolding, ΔG_u_ are defined as

$$\mathrm{Tetramer}:K_{u}={\left[U\right]}^{4}/[N_{4}]$$ (13)

$$=4\;{P_{t}}^{3}{f_{u}}^{4}/(1-f_{u})$$ (14)

$$\Delta G_{u}=-\mathrm{RT}\;\ln\;K_{u}\;$$ (15)

where P_t_ is the total concentration protein monomer and f_u_the fraction of denatured protein. At any given denaturant concentration [D], f_u_ was calculated as the ratio

$$f_{u}=(I-I_{n})/(I_{u}-I_{n})$$ (16)

where I is the fluorescence intensity at a given denaturant concentration and I_n_ and I_u_ are the same corresponding to the native (n) and denatured (u) states of the protein respectively. ΔG_u_^H^_2_^0^ and *m* were determined from the plot of the ΔG_u_ versus denaturant concentrations from the following equation.

$$\Delta G_{u}=\Delta {{{G_{u}}^{H}}_{2}}^{0}-m[D]$$ (17)

Free energy of stabilization for erythroid and non-erythroid spectrin were also estimated by fitting the raw data without converting to the fraction of unfolded proteins according to Street et al. [59, 60]. Equilibrium unfolding induced by urea and GuHCl were related to equilibrium constant for unfolding assuming a population-weight average signal of the native (Y_N_) and denature state (Y_D_) states.

$$Y_{\mathrm{obs}}=f_{N}Y_{N}+f_{D}Y_{D}=(1/1+K_{u})\;Y_{N}+(K_{u}/1+K_{u})\;Y_{D}$$ (18)

Where f_N_ and f_D_ is the fraction of native and denature protein, K_u_ represents the equilibrium constant for folding Y_N_ and Y_D_ are assumed to be linear dependence of denaturant, are represent represented as: Y_N_ = a_N_ + b_N_ [Denaturant] and Y_D_ = a_D_ + b_D_ [Denaturant]. The equilibrium constant for folding is related to the reaction free energy by the standard formula:

$$K_{u}=\exp^{\left(-\Delta \mathrm{Gu}0/\mathrm{RT}\right)}$$ (19)

The two state model was fit to the data obtained from fluorescence and CD signals and were expressed by combining equation 1 and 2, Y_obs_ = a_N_ + b_N_ [Denaturant] + Y_D_ = a_D_ + b_D_ [Denaturant] + exp ^(−ΔGu0/RT)^ / 1 + exp ^(−ΔGu0/RT)^. Thermodynamic parameters were estimated using the linear extrapolation method, in which the change in free energy of unfolding varied linearly with the denaturant concentration (equation 17).

## Results

### Fluorescence study of unfolding of erythroid and non-erythroid spectrin

**Figure 1:**
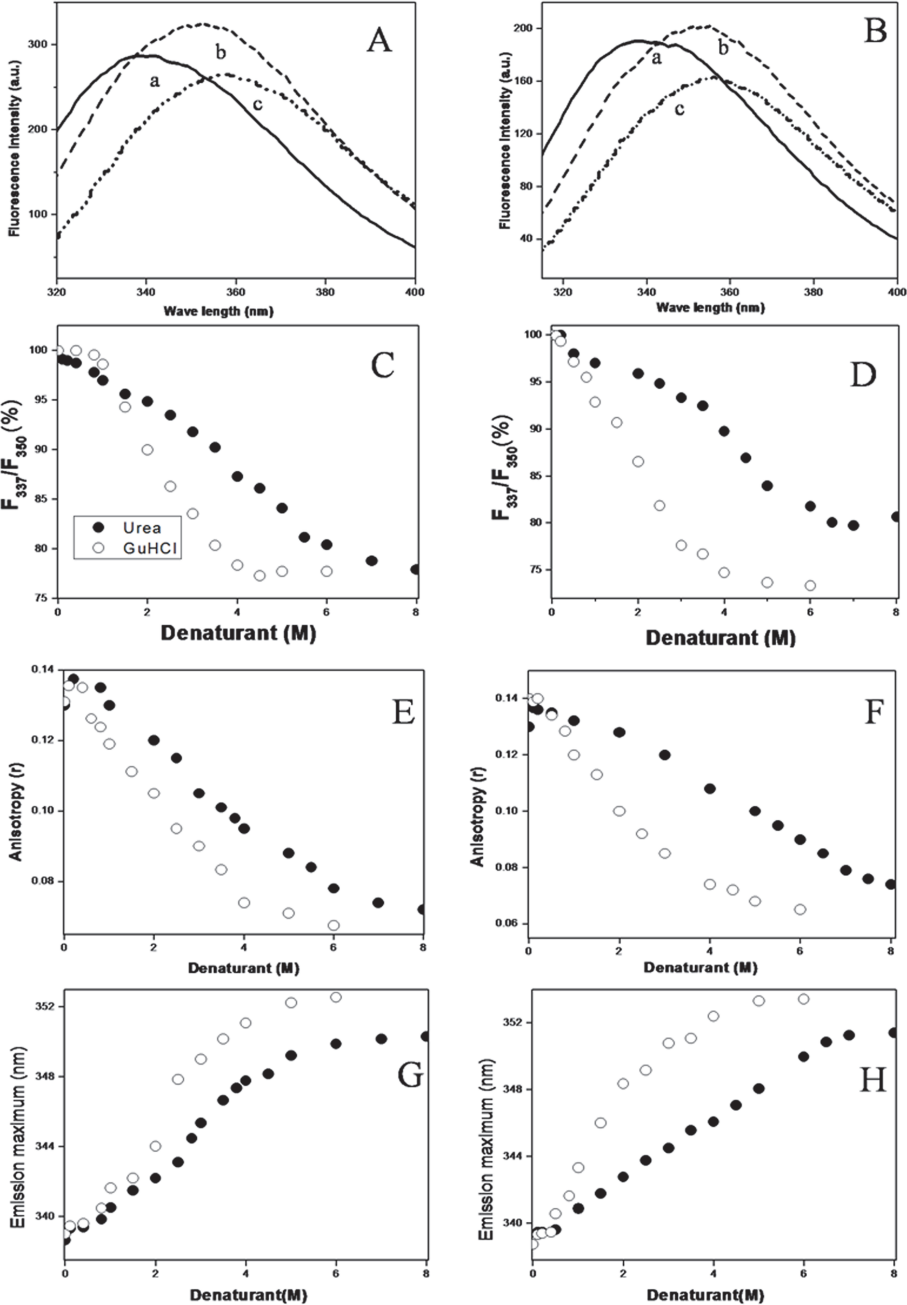
Structural changes in erythroid, nonerythroid spectrin in presence of increasing concentrations of urea and GuHCl monitored by fluorescence spectroscopy at pH 8.0 and 25°C. Fluorescence emission spectra of (A) erythroid spectrin and (B) nonerythroid spectrin are shown for the native (a), the unfolded protein in 8M urea (b) and the unfolded protein in 6M GuHCl (c). Changes in the ratio of intensities at 337nm and 350nm of tryptophan fluorescence (F_337_/F_350_) against the denaturant concentrations are shown in (C) for erythroid spectrin and (D) for non-erythroid spectrin. The data are normalized by taking (F_337_/F_350_) of native protein as 100%. Urea and GuHCl induced unfolding are shown by changes in steady state fluorescence anisotropy (r) in (E) erythroid and (F) nonerythroid spectrin and by the changes in emission maxima (λ_max_) in (G) erythroid and (H) nonerythroid spectrin respectively.

The spectral parameters of tryptophan, such as intensity, wavelengths of maximum emission (λ_max_) and spectral shape are dependent on the microenvironment in and around the Trp residue and has been extensively used to obtain information on the structural and dynamical properties of the protein [61, 62]. For monitoring denaturant induced structural and dynamical properties of spectrin, all experiments are performed at 25°C after incubation with the denaturants for 1 hour, during which no proteolytic cleavage of the protein occurred, shown in S1 Fig.. We have studied the change in the ratio of intensities at 337 nm and 350 nm and emission maxima (λ_max)_ of Trp fluorescence in presence and absence of urea and GuHCl in erythroid and non-erythroid spectrin (Fig. 1). The λ_max_ appeared at 338nm and 338.5nm respectively for both erythroid and non-erythroid spectrin indicating buried tryptophan residues in the folded protein [51]. In absence and presence of submolar concentrations, up to 0.8M, of urea / GuHCl, tryptophan fluorescence is quenched with no shift in the λ_max_. In presence of urea above 6M, erythroid spectrin shows an enhancement of intensity with a shift in λ_max_ to 350–351 nm and from 338 nm indicating exposure of tryptophans upon complete unfolding [62]. In presence of GuHCl above 4M, λ_max_ appeared at 350 nm - 353 nm, associated with a decrease in intensity for both forms of spectrin upon denaturation.

From the measured dependence of relative fluorescence intensity (*F_337_* /*F_350_*) and the λ_max_ as a function of the denaturant concentration we observe that the unfolding transition curve for both erythroid and nonerythroid spectrin are sigmoidal in nature (Fig. 1). The value of urea and GuHCl concentrations at the half the completion of the transition, was found to be [urea]_1/2_ = 3.9M and 4.0M and [GuHCl]_1/2_ = 2.0 M, from (F_337_/F_350_) and λ_max_ respectively. They also indicate the unfolding to go through a reversible two state transition of N_2_«2D or N_4_«4D for both the proteins, without formation of any intermediate state (Fig. 1 and Table 1). Tetrameric erythroid spectrin also showed denaturant induced sigmoidal transition similar to both froms of spectrin shown in the supplementary material (S2 Fig.). The value of urea and GuHCl concentration at the half of the completion of transition was found to be 3.8 M and 2 M respectively (Table 1), which indicates that tetrameric erythroid spectrin also unfolds in concerted manner without formation any intermediate state.

**Table 1:** Values of urea and GuHCl concentration at half completion of unfolding transition for erythroid and non-erythroid spectrin determined by CD and fluorescence spectroscopic measurements at pH 8.0 and 25°C.

**Protein**	**Probe**	**[Urea]_1/2_(M)**	**[GuHCl]_1/2_(M)**
Erythroid spectrin (Dimer)	Intensity ratio	3.9	2.0
	f_u_	4	2.1
	Anisotropy	3.9	2.0
	λ_max_	3.9	2.0
	ANS	3.8	2.1
	[θ]_222_	4	1.9
Non-erythroid spectrin (Tetramer)	Intensity ratio	4.0	2.1
	f_u_	4.1	2.1
	Anisotropy	4.0	2.0
	λ_max_	4.0	2.0
	ANS	3.9	2.0
	[θ]_222_	4.0	2.0
Erythroid spectrin (Tetramer)	Intensity ratio	4.0	2.0
	f_u_	3.8	1.9

Steady state fluorescence anisotropy (r) is commonly used to study the structural flexibility of proteins [51]. The anisotropy values of erythroid, non-erythroid spectrin as a function of urea concentration are also shown in Fig. 1. In presence of very low concentration of urea the anisotropy value increases and then remains unchanged up to a concentration of 1M. Anisotropy value drops significantly from 0.13 to 0.075 when urea concentration increased from 1M to 6M and above. Unfolding profile observed for anisotropy versus concentration of the denaturants followed similar pattern to those seen in case of (F_337_/F_350_) and λ_max_ for both the proteins, also indicating two state transition with [urea]_1/2_ = 3.9M and 4.0M and [GuHCl]_1/2_ = 2.0 M respectively, for both erythroid and nonerythroid spectrin (Table 1). This clearly suggests that the addition of denaturants above a threshold concentration induce loss of overall structure and conformation in both erythroid and non-erythroid spectrin and increase in the freedom of mobility of the large number of Trp residues in the unfolded proteins. S3 Fig. shows the data of change of anisotropy against denaturant concentrations during refolding process, indicating both unfolding and refolding processes of dimeric erythroid and tetrameric non-erythroid spectrin to be reversible in nature as also observed by others previously [63, 64, 65].

### Probing the secondary structure by far-UV CD spectroscopy

**Figure 2:**
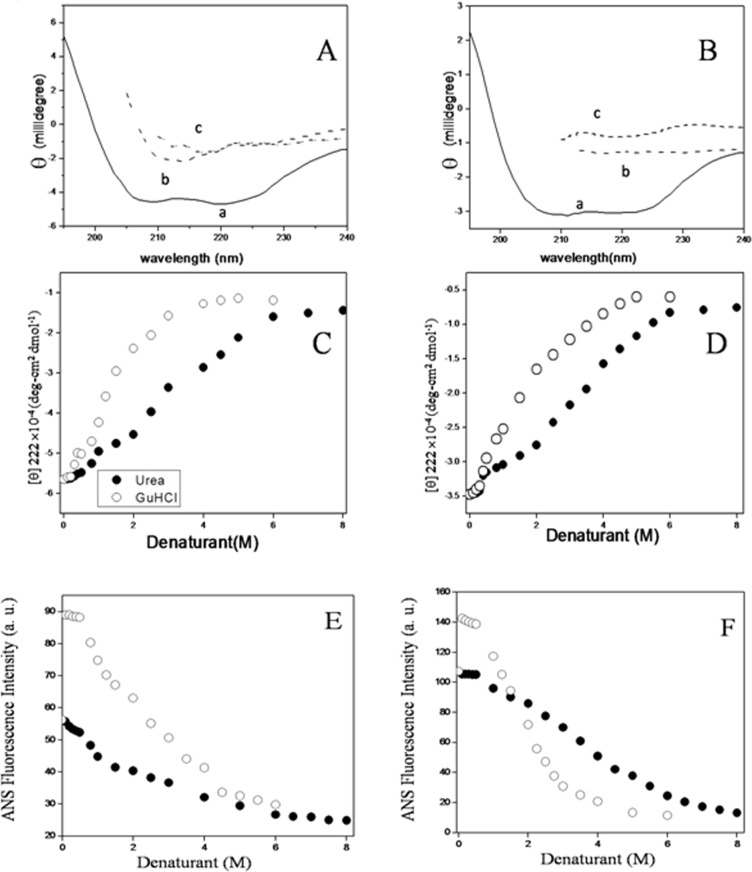
Denaturation of erythroid and nonerythroid spectrin monitored by CD spectroscopy. CD spectra of (A) erythroid spectrin and (B) nonerythroid spectrin showing the native (a) and the unfolded proteins in 8M urea (b) and 6M GuHCl (c). Urea and GuHCl induced changes in secondary structure of (C) erythroid spectrin and (D) nonerythroid spectrin by changes in ellipticity at 222 nm with increasing concentrations of the denaturants. Effects of urea and GuHCl on erythroid, non-erythroid spectrin (0.2 μM each) are shown by changes in ANS fluorescence at 470nm in (E) erythroid and (F) nonerythroid spectrin respectively.

Circular Dichroism spectroscopy is widely used in monitoring the conformation and stability of protein [66, 67]. Erythroid, non-erythroid spectrin display a far UV spectrum that have two minima at 208 nm and 222 nm which clearly indicate that both of them are typically α-helical protein which agree with previous studies, shown in Fig. 2 [46, 47, 48]. Plots of mean residue ellipticity at 222 nm ([θ]_222_) as a function of urea and GuHCl concentrations are shown in Fig. 2 for both erythroid and non-erythroid spectrin. Up to 1M urea, marginal increase in the negative value of ellipticity of native protein was observed. It then gradually decreased in presence of 2M till 6M urea and further decreased at concentration above 6M, from 100% to 29%, 25% respectively for both erythroid and non-erythroid spectrin. The GuHCl induced changes in the secondary structure of erythroid, non-erythroid spectrin also followed similar trend and transition profiles followed sigmoidal pattern with increasing concentration of the denaturants. At concentration higher than 4M GuHCl, the mean residue ellipticity decreased to 27% and 22% respectively for erythroid and non-erythroid spectrin. The [urea]_1/2_ was found to be 4.0M and [GuHCl]_1/2_ to be 1.9M for both the forms of spectrin respectively, which are also in good agreement with the fluorescence data, summarized in Table 1.

### Denaturant induced conformational change in ANS-bound spectrin

The emission spectra of ANS are very sensitive to environmental polarity. The emission intensity, quantum yield, and fluorescence lifetime substantially increase with large blue shift when bound to the hydrophobic surface of the protein. ANS is particularly used to characterize the folding intermediates, probing surface hydrophobicity and aggregation of proteins [68, 69, 70, 71]. In aqueous buffer, ANS shows λ_max_ at 520 nm, which upon binding to spectrin shifts to 470 nm with enhancement of fluorescence [21]. Fig. 2 shows that in presence small amount GuHCl ANS fluorescence intensity increases where as in presence of urea its almost remain same. In both dimeric erythroid (Fig. E) and tetrameric non-erythroid spectrin (Fig. 2F) small amount GuHCl increased the fluorescence intensity shown as overlap of both blank and filled circle but not in presence of urea. Further addition of both the denaturants makes the fluorescence intensity of spectrin-bound ANS in both forms of spectrin follow similar pattern as observed for some other proteins.

### Exploration of structural dynamics by Trp fluorescence quenching

**Figure 3:**
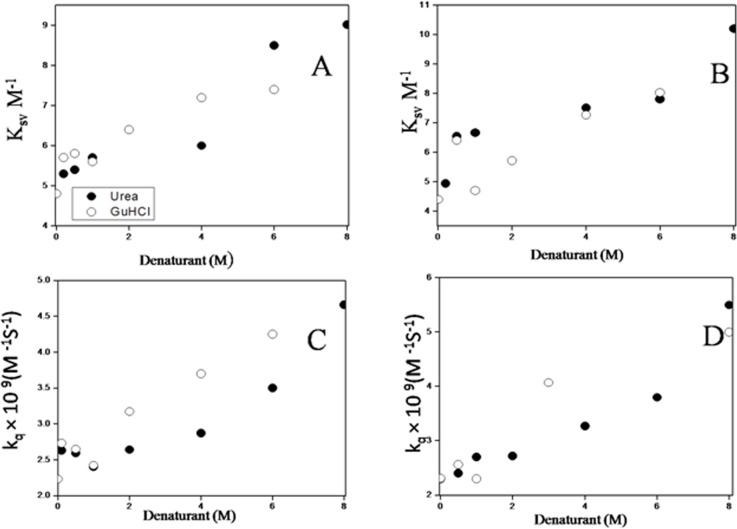
Acrylamide quenching of spectrin tryptophans showing plots of Stern Volmer constant (K_SV_) versus denaturant concentrations for (A) erythroid spectrin and (B) nonerythroid spectrin. Changes in bimolecular quenching rate constants (k_q_) versus denaturant concentrations are shown in (C) for erythroid and (D) for nonerythroid spectrin.

Measurement of quenching of tryptophan florescence by an external quencher is very useful to examine the conformation changes of proteins or peptides in sensing the exposure of the side chain of amino acid such as tryptophan to solvent [51]. Quenching experiments were carried out with erythroid, non-erythroid spectrin using a neutral quencher (acrylamide) and an anionic quencher (iodide ion) as elaborated our earlier work to probe conformational alterations in presence of the denaturants [52]. Stern-Volmer plots of erythroid and non-erythroid spectrin in presence and absence of different concentration of the denaturants indicate changes in the bimolecular quenching constant, K_SV_ evaluated from the slope of the plots. From the modified Stern-Volmer plot (Lehar-plot), the accessibility for acrylamide and iodide were evaluated. Few representatives Stern-Volmer plots of acrylamide and iodide quenching in the presence and absence of the denaturants are shown in S4 Fig.. S1 Table summarizes the acrylamide quenching parameters represented by *f_e_*, K_SV_ and the quenching rate constant k_q_ for spectrin in their respective native and denatured state. Variations of K_SV_ and k_q_ with the denaturant concentrations showed both to increase with increasing concentrations of the denaturants due to larger quencher accessibility of the exposed Trp residues, shown in Fig. 3. The k_q_ values were determined from the slope of the plots of τ_0_/τ against the acrylamide concentration elaborated previously [52]. K_SV_ came out to be 4.6±0.2 M^−1^ and 4.4±0.2 M^−1^ for the acrylamide quenching of native erythroid and non-erythroid spectrin respectively which increased significantly in 8M urea to 8.7±0.4 M^−1^ and 10.2±0.4 M^−1^. Similar increase to 7.4±0.3 M^−1^ and 8.0±0.4 M^−1^ respectively, were found in the presence of 6M GuHCl indicating exposure of tryptophan residues during unfolding (S1 Table). In case of quenching with iodide K_SV_ increased from 2.1±0.2 and 1.7±0.2 to 3.2±0.3 and 2.98±0.2 in 8M urea and 4.01±0.2 and 3.4 ± 0.3 in 6M GuHCl for erythroid, non-erythroid spectrin respectively (S2 Table). As a control experiment similar quenching studies were also carried out on the model tryptophan NATA. K_SV_ (16.5±0.3) remained unaltered in presence and absence of GuHCl and urea further indicating the change in K_SV_ to be specific for erythroid, non-erythroid spectrin [52].

S5 Fig. shows the variation of mean lifetime of Trp as a function of denaturant concentrations indicating a steady decrease with increasing urea and GuHCl. The mean lifetime values in native erythroid and non-erythroid spectrin were found to be 3.82 ns, 3.80 ns respectively which is in perfect agreement with the previously reported value [47, 48, 52].

### DLS measurements of spectrin under denaturing conditions

**Figure 4:**
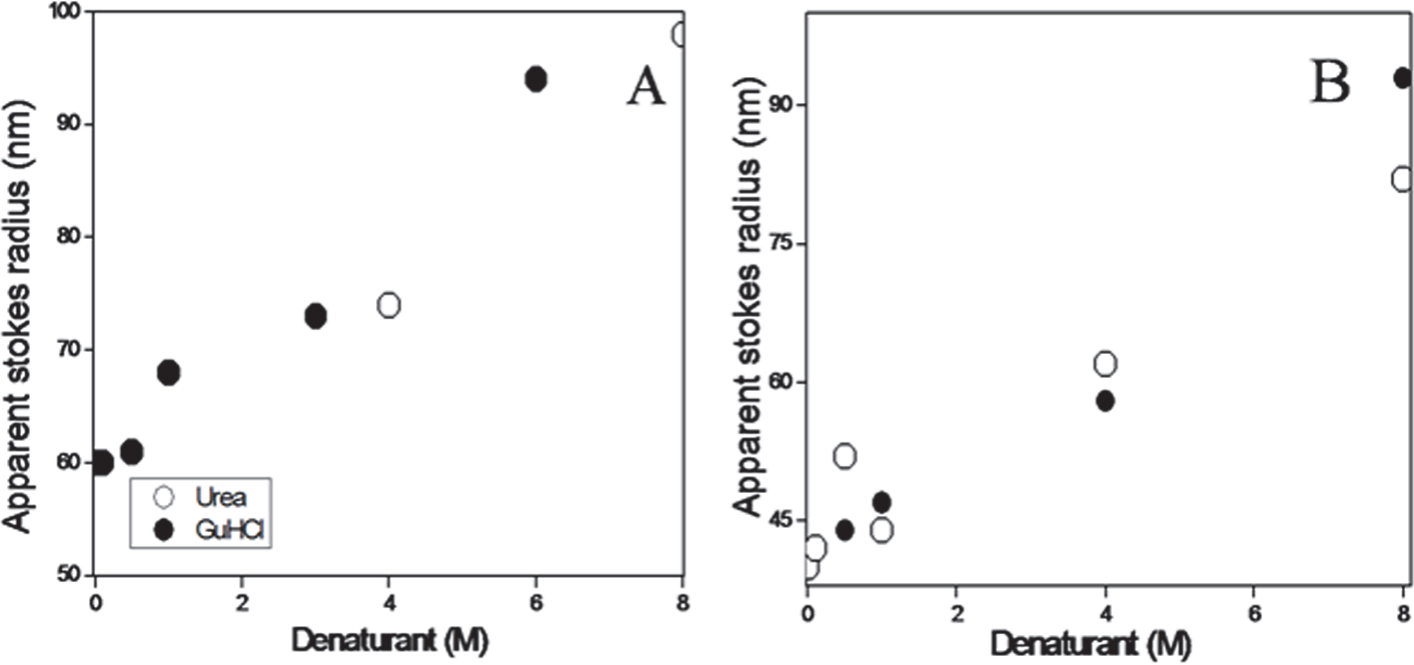
Changes in apparent hydrodynamic radius of the erythroid (A) and nonerythroid spectrin (B) with increasing concentrations of urea and GuHCl.

**Figure 5:**
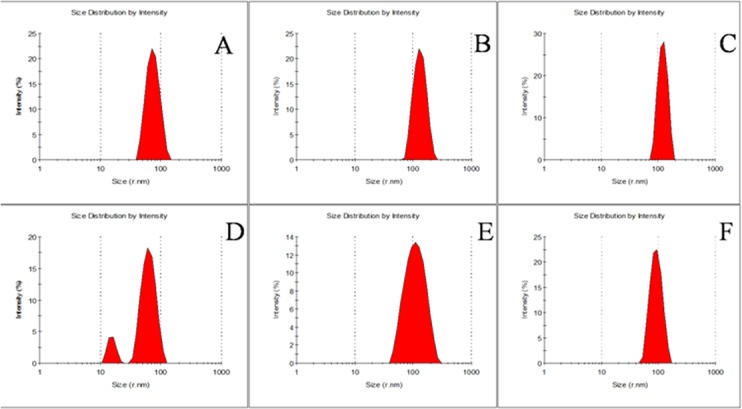
Compactness of erythroid and nonerythroid spectrin under different conditions. Histograms show size distribution of spectrin species obtained by DLS measurements of erythroid spectrin in (A) native; (B) unfolded in 8M urea and (C) unfolded in 6M GuHCl and of non-erythroid spectrin in (D) native; (E) unfolded in 8M urea and (F) unfolded in 6M GuHCl.

DLS measurements were carried out to monitor the changes in size of the erythroid and nonerythroid spectrin in presence and absence of urea or GuHCl. The changes in the apparent hydrodynamic radii (R_h_) of spectrin against increasing concentrations of urea and GuHCl, are shown in Fig. 4. Surprisingly, the apparent hydrodynamic radii of dimeric erythroid and tetrameric nonerythroid spectrin were estimated to be 60±10 nm and 40±5 nm respectively, which are in good agreement with Budzynski and co-workers [55, 56]. The hydrodynamic radius of both spectrin remained almost same up to 1M urea and beyond that the particle size significantly increased with denaturant concentration up to 4M. At a concentration of 8M urea, the hydrodynamic radii of completely unfolded erythroid and nonerythroid spectrin were 95±10 nm and 85±7 nm respectively, shown in Fig. 5. The hydrodynamic radii increased by the factor *f* = R_h unfold_ / R_h nat_ of 1.6 and 2.0 respectively for erythroid and nonerythroid spectrin in 6M GuHCl. Previous reports showed that the hydrodynamic stokes radius of lysozyme and RNase A increased by a factor 1.45 and 1.37 respectively in GuHCl induced denatured state [72, 73, 74]. The hydrodynamic radius (R_h_ ) measured for BSA in urea and GuHCl are shown in S6 Fig., used as control. The DLS profile of native BSA shows two peaks with hydrodynamic radii of 3.8±0.2 nm and 80 nm respectively, corresponding to the monomeric BSA and that of oligomeric BSA. On the other hand, the completely unfolded BSA showed a hydrodynamic radius of 8.5±0.5 nm in higher than 7M urea and 9.5±0.5 nm in higher than 3M GuHCl which is comparable to the previously obtained value [75].

### Energetics of urea and GuHCl induced conformational stability of spectrin

**Figure 6:**
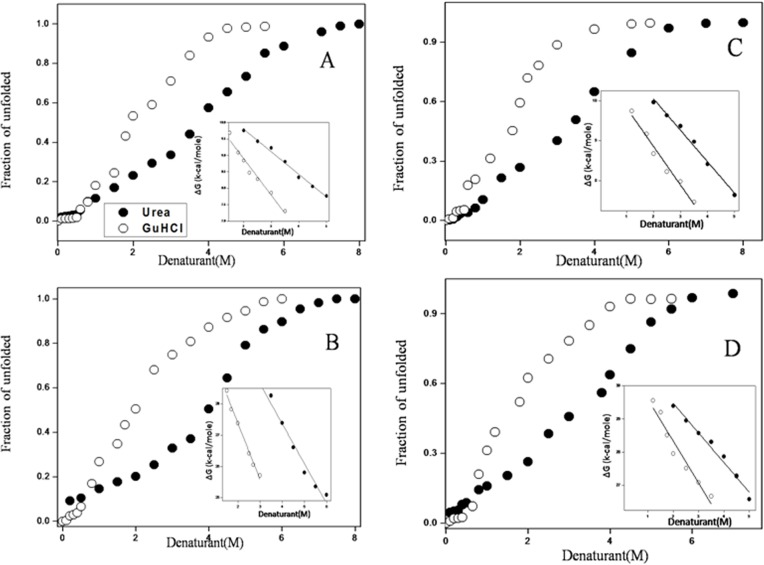
Unfolding curves for erythroid (A) and nonerythroid spectrin (B) are followed by tryptophan fluorescence intensity. Insets show the linear free energy extrapolation curve with respect to increasing concentration of the denaturant concentrations. The ΔG_D_^H20^ was obtained from the intercept on Y-axis by using linear extrapolation. Similar unfolding curves are followed by CD spectroscopy, shown for (C) erythroid (D) non-erythroid spectrin. Insets show the linear free energy extrapolation curve with respect to increasing concentration of the denaturant concentrations. The ΔG_D_^H20^ was obtained from the intercept on Y-axis by using linear extrapolation.

It has been established that urea and GuHCl induced unfolding of erythroid and non-erythroid spectrin takes place in a reversible and cooperative manner without the formation of any stable intermediate. The unfolding process was followed with four different spectroscopic parameters. The pattern of unfolding equilibrium curve constructed with increasing denaturant concentrations indicates large conformational changes in the secondary and tertiary structures. Assuming a two state transition model, the unfolded fraction of erythroid and non-erythroid spectrin for urea and GuHCl were obtained to determine the free energy of stabilisation (ΔG_u_^H^_2_^0^). ΔG_u_ values were obtained by linear extrapolation in absence of the denaturant following equation (17) [57, 58]. Figs. 6 and S7–S8 show the unfolding curves against concentrations of the two denaturants for both erythroid and non-erythroid spectrin in terms fraction of unfolded structure (f_u_). From such profiles [urea]_1/2_ was found to be 4.0M and 4.1M for erythroid and non-erythroid spectrin respectively. Insets of Fig. 6 and S7 show the change in free energy of unfolding for erythroid and non-erythroid spectrin as a function of urea concentration. The extrapolated standard free energy, (ΔG_u_^H^_2_^0^) at zero urea concentration has been ∼ 11 kcal/mole and ∼31 kcal/mole respectively for erythroid and non-erythroid spectrin. From the GuHCl induced denaturation curve the calculated free energy of stabilisation (ΔG_u_^H^_2_^0^) in absence of denaturant values are ∼ 10.5 kcal/mole and ∼ 30 kcal/mole (Table 2) for erythroid and non-erythroid spectrin at 25°C and pH 8.0. We have also measured stability of tetrameric erythroid spectrin. Inset of S2 Fig. shows the change in free energy of unfolding for tetrameric erythroid spectrin as a function of denaturant concentrations. The extrapolated free energies for tetrameric erythroid spectrin at zero denaturant concentration were similar to tetrameric non-erythroid spectrin. As the stability curve does not have well defined base line, we also measured the free energy of stabilization according to the method of Street et al (S8 Fig.) [59, 60]. The free energies of stabilization for dimeric and tetrameric spectrin are ∼ 9.7 kcal/mole and ∼ 28 kcal/mole respectively which are good agreement with the data ∼ 11 kcal/mole and ∼ 30 kcal/mole respectively summarized in Table 2. Using both methods the estimated free energy of stabilization are similar and Cm and m values are in excellent agreement with previous results [43]. Free energy of stabilization of a protein is determined by multiplying m value with the transition mid-point. However, the dimeric and tetrameric proteins do not regularly follow this rule [76, 77, 78]. The *m* values, which are related to changes in solvent surface accessibilities, from the native state to denatured state, is greater for GuHCl induced unfolding compared to urea for both forms of spectrin. Based on the higher *m* value and lower [D]_1/2_ GuHCl acts as a stronger denaturant than urea for the two membrane skeletal proteins.

**Table 2:** Thermodynamic parameters of urea and GuHCl induced unfolding of erythroid and non-erythroid spectrin as determined by equilibrium denaturation experiment.

**Condition**	**Protein**	**Probe**	**DG^u^_H2O_(Kcal/mole)**	**m _U-N_(Kcal mole^−1^ M^−1^)**
Urea	Erythroid spectrin (Dimer)	Fluorescence	11.16	−0.80
		CD	10.86	−0.78
		ANS	10.66	−0.75
	Non-erythroid spectrin (Tetramer)	Fluorescence	32.46	−1.40
		CD	31.25	−1.24
		ANS	31.70	−1.14
	Erythroid spectrin (Tetramer)	Fluorescence	30.40	−1.23
GuHCl	Erythroid spectrin (Dimer)	Fluorescence	10.81	−1.11
		CD	10.79	−1.06
		ANS	10.76	−1.12
	Non-erythroid spectrin (Tetramer)	Fluorescence	30.99	−1.80
		CD	30.84	−1.25
		ANS	31.31	−1.20
	Erythroid spectrin (Tetramer)	Fluorescence	29.86	−2.03

## Discussion

Unfolding of oligomeric proteins is often complex and different compared to small, monomeric proteins. The protein oligomers, the monomers in the native form and the unfolded peptides coexist whereas the unfolding mixture contains only the native monomers and the unfolded peptides during unfolding of monomeric proteins [79, 80, 81]. The present study is undertaken to improve our understanding on the molecular mechanism of unfolding of a large, multidomain protein, spectrin since many important proteins function in their oligomeric from. Spectrin, is composed of large number of three helical tandem repeat motifs and this homologues repeats arranged in a zigzag fashion, make up most of the spectrin molecule. The worm-like protein maintains its elongated shape, ability to change its length at different ionic strength, its inherent flexibility and ability to rapidly renature from unfolded state. Several studies on the conformational and structural stability have been done with one or two repeat motifs of spectrin [35, 36, 37, 38, 39]. Kinetic studies on denaturation of two tandem repeat motifs of spectrin indicated the unfolding to be cooperative and the stability of such repeats attributed to specific interactions between the neighboring as well as non-natural domains inducing stabilization [37]. The structural disorder, seen in the C-terminal tandem repeat motifs of spectrin was found to be due to lack of a stretching helix [36]. Calorimetric studies indicated a single spectrin to be more thermally stable than the intact spectrin heterodimers or isolated subunits [35]. The stability of intact spectrin is important in the maintenance of the skeletal architecture inside cell. This is also implicated in some genetic hematological disease such as hereditary pyropoikilocytosis, hereditary elliptocytosis and hereditary spherocytosis, associated with mutation of spectrin [17, 18, 82, 83]. In the absence of elaborate studies on the stability of intact spectrin, we’ve undertaken the present study on the comparison of stability of both forms of intact spectrin, between dimeric and tetrameric as well as the erythroid and the nonerythroid forms of spectrin. The wavelength of maximum emission at 338±1 nm and a high anisotropy value of 0.13±0.01 for both erythroid and nonerythroid spectrin in absence of the denaturants indicate that the Trps in spectrins are shielded from bulk water, however, are not located in the hydrophobic core of the proteins. The red-sift in the λ_max_, decrease in anisotropy and mean lifetime indicated unfolding of both forms of spectrin increasing the exposure of Trp residues to polar environment. From the dependence of fluorescence and far UV circular dichroism with the denaturant concentrations, we observed that both dimeric erythroid and tetrameric spectrin unfold following a classical two-state transition without the formation of any stable intermediate. Both from CD spectroscopy and our earlier work on wavelength selective fluorescence show loss of secondary structure from 100% to about 25% in presence 8M urea and 6M GuHCl, clearly indicating that both forms of denatured proteins still retain some residual secondary and tertiary structures [45].

It has been previously shown that quenching of tryptophan fluorescence could be used to study the conformational changes in spectrin and its subunits [52, 62]. Neutral quenchers like acrylamide could penetrate into a hydrophobic interior of the folded protein and quench even partially buried tryptophan residues, on the other hand charged quenchers like iodide access only the surface exposed tryptophan residue. Acrylamide quenching yielded the K_SV_ values of 4.6±0.2 M^−1^ and 4.4±0.2 M^−1^, on the other hand, iodide quenching yielded the K_SV_ of 2.1±0.2 and 1.7±0.3 for both forms of spectrin respectively. Quenching experiments indicate most the tryptophan residues to remain in either conserved of moderately conserved region in both erythroid and non-erythroid spectrin [43, 44]. The bimolecular quenching constants (k_q_) also led to similar results. In presence of 8M urea or 6M GuHCl, quenching efficiencies of both the quenchers increase markedly, indicating the exposure of the masked and buried tryptophans upon unfolding. With increasing concentration of urea / GuHCl, the quenching constants increased after a marginal decrease in submolar region upto 0.8 M in both forms of spectrin indicating a probable unzipping of the spectrin subunits without complete dissociation. The quenching data also indicated distinct structural differences between the unfolded proteins induced either by 6M GuHCl or by 8M urea [52].

The hydrodynamic study on spectrin was first reported by Ralston and co-workers in 1976 [84]. Using ultracentrifugation method they showed that spectrin molecules are long and flexible. Using total intensity light scattering, Elagaster and coworkers later found the radius of gyration of dimeric spectrin to be 22 nm which was doubled in low ionic strength [85]. DLS has been extensively used to investigate the size and shape of the intact spectrin and their recombinant fragments [55, 56]. It has been shown that the hydrodynamic radii of spectrin molecules fluctuate between 22–30 nm in presence of high salt but in low salt its undergoes molecular extension [86, 87]. Non-erythroid spectrin, on the other hand always remains as tetramer under physiological salt concentration. Our DLS data are also in very good agreement with those of Budzynski and co-workers, shown in Figs. 4 and 5 [55]. Completely unfolded spectrin showed the molecular size of both erythroid and non-erythroid spectrin to increase by a factor of 1.5–2.0. Similar study with the globular BSA showed the same increase by a factor of 2.4 to 2.5.

Spectrin has large number of hydrophobic stretches in its polypeptide sequence, which can bind hydrophobic ligands and phospholipids and cause quenching of tryptophan fluorescence of protein [46, 48]. The hydrophobic probe 1,8-ANS has been extensively used as probe to measure the hydrophobicity of protein surface to gain more information about stability of spectrin in presence of the denaturants [71]. The fluorescence intensity of spectrin-bound ANS decreased as a function of increasing concentrations of urea/GuHCl and showed sigmoid like transition curve, ruling out the existence of any intermediate state in both the erythroid and nonerythroid spectrin (Fig. 2).

Denaturants acts as both stabilizing and destabilizing agent and their effects arise from the conformations of protein. A clear understanding of their mode of action is however, lacking. In subdenaturing concentration of denaturants, proteins get stabilized by binding of denaturants its polypeptide chain. Previous studies indicate that polyfunctional interaction between proteins groups and denaturants are responsible for such stabilization [88, 89, 90]. The interaction of the denaturant molecule with different groups of protein through noncovalent bonding can established nonspecific network of intramolecular interaction. Such denaturant mediated crosslinking of different part of the protein leads to a decrease of motional freedom. Existing X-ray data on lysozyme and ribonuclease A show that a significant decrease of B-factor of side chain and backbone atoms in presence of low concentration of denaturant compare to native state protein in absence of denaturant [88, 91, 92]. The present study also indicates that at submolar concentration of the denaturants, spectrin gets stabilized and undergoes changes in the quaternary structure alone without change in its secondary and tertiary structure. Fluorescence anisotropy offers a convenient method for obtaining information rotational dynamics of the fluorophore. The rotational freedom of both dimeric erythroid and tetrameric non-erythroid spectrin decreased in presence low concentration denaturant leading to increase in anisotropy from 0.13 to 0.138. The anisotropy decreased to 0.06 when the protein was completely denatured in either 8M urea or 6M GuHCl. Quenching of tryptophan fluorescence by acrylamide yielded a value 4.6 and 4.4 for K_SV_ in the native state of both proteins [47, 52]. In presence of low concentrations of the denaturants the quenching efficiency decreases markedly pointing to a lesser exposure of the tryptophan residues. This also confirms that the tryptophans are in more hydrophobic environment indicating lesser accessibility compared to the native protein. All these data indicate that the major effect of low concentration of the two denaturants, on the Trp anisotropy and quenching parameters are due to binding of denaturant in the both side chains and the backbone of atoms of the proteins. Similar effects are observed when the fluorescence intensity of spectrin-bound ANS markedly increases in presence of low GuHCl concentration and then decrease with increasing concentration of denaturants. Such increase of fluorescence intensity of hydrophobic dye and anisotropy may also be indication of protein aggregation or transformation of the protein to a folding intermediate like molten globular state when denaturation is caused by GuHCl. However, in this case the effect is especially binding GuH ^+^ to the negative surface of the protein molecule and it is accompanied only by the increase in fluorescence intensity but not an increase in the intensity of dynamic light scattering. The pI of spectrin is 5.5 and therefore spectrin molecule is negatively charged in neutral pH. With an increase in number of GuH^+^ ions bound to spectrin, the number of positively charged groups increases to neutralize the surface charges leading to increase in ANS fluorescence. Upon further increase in GuHCl concentration the number of positively charged groups exceeds the negatively charged groups, leading to decrease in ANS fluorescence. Similar effect is also observed in our earlier studies with spectrin-bound Prodan, in presence of low concentrations of the denaturant [52]. All these data indicate that the major effect of low concentration of the two denaturants, on the Trp anisotropy and quenching parameters are due to changes in the quaternary structure originating from a immobilized microenvironment where the spectrin subunits remain strongly associated without effecting its tertiary and secondary structure. The origin of the quaternary structural changes is then due to a possible unzipping of the subunits in the super coiled rod domain of both forms of the proteins also altering the overall flexibility of the membrane skeletal protein which may take place in the membrane skeleton under changes in physiological condition.

It has been shown previously by Marchesi and coworkers that fluorescence polarization technique can be used for monitoring quaternary and tertiary structural changes of spectrin molecule [64, 93]. They also showed that urea increase tryptophan mobility at all temperature but changes caused by urea in the 2–4M concentration range are particularly interesting. Spectrin after incubation in 2–4M urea showed the steepest increase tryptophan mobility, resulting in the dissociation of the subunits to monomer which could be separated using DEAE cellulose chromatography. The concentration of urea at 3M is critical, lower concentration of urea does not dissociate the subunits completely but still higher concentration of urea cause too much unfolding of the chains. Our results on ratio of fluorescence intensity, emission maxima, ANS fluorescence intensity and changes in mean residue ellipticity indicate that both dimeric and tetrameric erythroid and tetrameric non-erythroid spectrin undergoes secondary and tertiary structural changes between 2 to 6 M in urea and 1 to 4M GuHCl. The major structural transition occurs in urea between 2 to 4 M urea which is good agreement with Marchesi and co-worker [64]. Tryptophan quenching and ANS fluorescence data show that presence of submolar chemical denaturants induces quaternary structural change in spectrin without causing complete dissociation of subunits. Higher concentrations of the denaturants caused considerable changes in the secondary and tertiary structure of spectrin. We also show that the presence of residual structure that remains even after spectrin is treated with denaturing concentrations of the denaturants can be considered as a hallmark of a cytoskeletal protein whose main function is to provide a stable scaffold to the cell membrane.

Actual mechanism of protein denaturation by chaotropic agents (urea or GuHCl) is still not clear. It is postulated that both have common features for protein denaturation. Both urea and GuHCl interact with the backbone amide group and polar side chains forming multiple hydrogen bonds and also cause an increase of surface pressure, leading to salting out from aqueous phase [94, 95]. Urea and GuHCl have very similar structure but in some cases difference in action between them is attributed to the ionic nature of GuHCl, the later being an electrolyte with pK_a_ of about 11. At normal pH the GuHCl remain in fully dissociated from GuH^+^ and Cl^−^. It is assumed that GuH^+^ is easily absorbed on the protein surface due its hydrogen bonding ability and can also weaken the electrostatic interaction. Such a mechanism can explain differential denaturation by urea and GuHCl. Even at submolar concentrations, both the denaturants can form multiple hydrogen bonds; participate in van der Waals interactions with the polypeptide backbone and the side chain groups of the protein leading to restriction of the motion [94, 96, 97].

**Figure 7:**
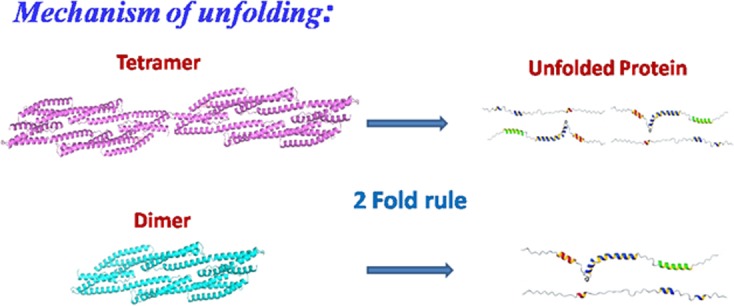
Schematic diagram on the mechanism of unfolding of dimeric and tetrameric spectrin.

Pace and co-workers first pointed out that unfolding of most monomeric globular protein in GuHCl is found to be 2 times effective than urea [98, 99]. This rule /is found to be valid only for mesospheric globular proteins where unfolding occurs in a concerted two step pathway. On the other hand, multimeric proteins are not generally found to obey this rule, although their susceptibility to GuHCl induced denaturation has been known [100, 101]. On the basis of equilibrium denaturation studies of dimeric erythroid and tetrameric non-erythroid spectrin, we are reporting for the first time that large, multi-domain, worm like membrane skeletal proteins could also obey two-fold rules as reported by Pace for small globular proteins (Fig. 7). X-ray diffraction studies with a single repeat domain of spectrin revealed large number of salt bridges between the three helix bundle and on the exterior surface of the protein, explaining the reason for weaker resistance against GuHCl through charge-charge interactions [27]. Large number of salt bridges in the exterior surface is also responsible for high thermal stability of such of both forms of spectrin.

Most of the earlier studies on equilibrium unfolding of erythroid and non-erythroid spectrin were performed using one or two repeat motifs of spectrin [35, 37, 38, 40]. Earlier studies with erythroid α-spectrin, chicken brain α-spectrin, recombinant α-16 and intact dimeric erythroid spectrin revealed comparable denaturation profile with similar [D]_½_ [43]. In this work we have compared conformational stabilities of intact dimeric and tetrameric erythroid and tetrameric non-erythroid spectrin, using free energy of unfolding in presence of the two denaturants at 25°C. The position of equilibrium unfolding transition curves, constructed by fluorescence, DLS and CD spectral parameters, were comparable in both forms of spectrin. The remarkable agreement between the changes in free energy during urea and GuHCl induced denaturation reveals that the systems remain in thermodynamic equilibrium independent of the denaturant used. Lower m values in presence of both urea and GuHCl indicates less exposed hydrophobic surface to the solvent in the unfolded erythroid dimeric spectrin, compared to the tetrameric nonerythroid spectrin [94]. The higher stability of both dimeric and tetrameric erythroid and tetrameric nonerythroid spectrin compared to the individual one or two repeat motifs in tandem, is due to the presence of larger number of helix-helix interaction between neighbouring domain, which decreases the unfolding rate but favours refolding rate. This activity has been attributed in intact spectrin due presence of large linking helix between two helix. Higher thermal stability of both repeat motifs and intact nonerythroid spectrin than the erythroid spectrin was shown previously on the basis of T_m_ (mid-point of melting). Due to greater rigidity of the intact spectrin and presence of 2–3 tandem repeats with helical linkers in between leads to elevation of T_m_ providing additional stability to its polypeptide sequence [39]. Similar value of [D]_1/2_ during unfolding of dimeric, terameric erythroid and tetrameric nonerythroid spectrin indicates both proteins to be equally stable. Thermodynamic data show that the tetrameric spectrin is more stable than the dimeric spectrin. MacDonald and co-workers have found six recombinant fragments consisting of two repeat motifs with a putative helical linker to be more stable than those with non helical linker [25]. They have also shown that pairs of more stable repeats in one dimer of a tetramer were located to the opposite side and the more stable pairs in a co-aligned dimer forms the hinge. Less stable fragments are located similarly opposite to each other on anti-parallel dimers of spectrin [38]. Clustering of such repeats in a spectrin tetramer gives spectrin its well-known flexibility. We have shown in this study that tetrameric spectrin is thermodynamically more stable and suitable for maintaining the proper architecture of the membrane skeleton and the overall morphology of the cell.

## Supporting Information

S1 Fig Representative gels after SDS-PAGE experiments of erythroid and non-erythroid spectrin in the presence and absence of urea. Lane (1) represent Molecular weight marker, lane (2) represent the protein after 1 hour incubation at 4°C, lane (3) at 25°C, lane 4, 5, and 6 represent erythroid spectrin after 1 hour incubation with 1M, 4M and 6M urea at 25°C. Lane 7, 8, and 9 represent the same for non-erythroid spectrin after 1 hour incubation with 1M, 4M and 6M urea at 25°C.
(TIF)

S2 Fig Changes of tryptophan fluorescence of tetrameric erythroid spectrin upon treatment with increasing concentrations of denaturants monitored by measuring the ratio of fluorescence intensity (F_337_/F_350_) upon excitation 295 nm, under different conditions. The data are represented as (A) the percentage of fluorescence taking the same for the native protein as 100% and (B) for the fraction of unfolded spectrin. Inset shows the linear free energy extrapolation curve with respect to increasing concentration of the denaturants. The ΔG_D_^H^_2_^0^ was obtained from the intercept on Y-axis by using linear extrapolation.
(TIF)

S3 Fig Refolding of dimeric erythroid (A) and tetrameric non erythroid (B) spectrin measured by tryptophan fluorescence anisotropy. (TIF)

S4 Fig Stern-volmer plot of quenching of tryptophan of erythroid spectrin (A and C), non-erythroid spectrin (B and D) by acrylamide and KI. The data points (a) for native; (b) for denatured spectrin in 8M urea and (c) in 6M GuHCl.
(TIF)

S5 Fig Mean lifetime of tryptophans in erythroid (A) and nonerythroid spectrin (B) in the presence and absence of the denaturants. (TIF)

S6 Fig Dynamic light scattering intensity distribution of BSA (A) in absence of denaturant (B) in 8M urea (C) in 6M GuHCl. The figure in the bottom panel represents hydrodynamic radii of BSA with different concentration of (D) Urea (E) GuHCl.
(TIF)

S7 Fig Chemical unfolding curves of (A) dimeric erythroid spectrin (B) tetrameric non-erythroid spectrin monitored by ANS fluorescence. Insets show the linear free energy extrapolation curve with respect to increasing concentration of the denaturant concentrations. The ΔG_D_^H20^ was obtained from the intercept on Y-axis by using linear extrapolation.
(TIF)

S8 Fig The linear free energy extrapolation curve with respect to increasing concentration of the denaturant concentrations obtained by fitting the raw data directly without converting to fraction of unfolded proteins. (TIF)

S1 Table The acrylamide quenching parameters of the tryptophan’s of erythroid, non-erythroid spectrin. K_SV_, f_e_, and k_q_ in the presence and absence of the denaturants.
(DOCX)

S2 Table The iodide quenching parameters of the tryptophan’s of erythroid, non-erythroid spectrin tryptophan’s K_SV_ and K_q_ in the presence and absence of denaturant. (DOCX)

S3 Table Thermodynamic parameters of urea and GuHCl induced unfolding of erythroid and non-erythroid spectrin by direct fitting of the raw data. (DOCX)
