# Supplementary figures and images for: Probing Conformational Stability and Dynamics of Erythroid and Nonerythroid Spectrin: Effects of Urea and Guanidine Hydrochloride
Source: PLoS One. 2015 Jan 24;10(1):e0116991. doi: 10.1371/journal.pone.0116991 (PMC4305312; doi:10.1371/journal.pone.0116991)

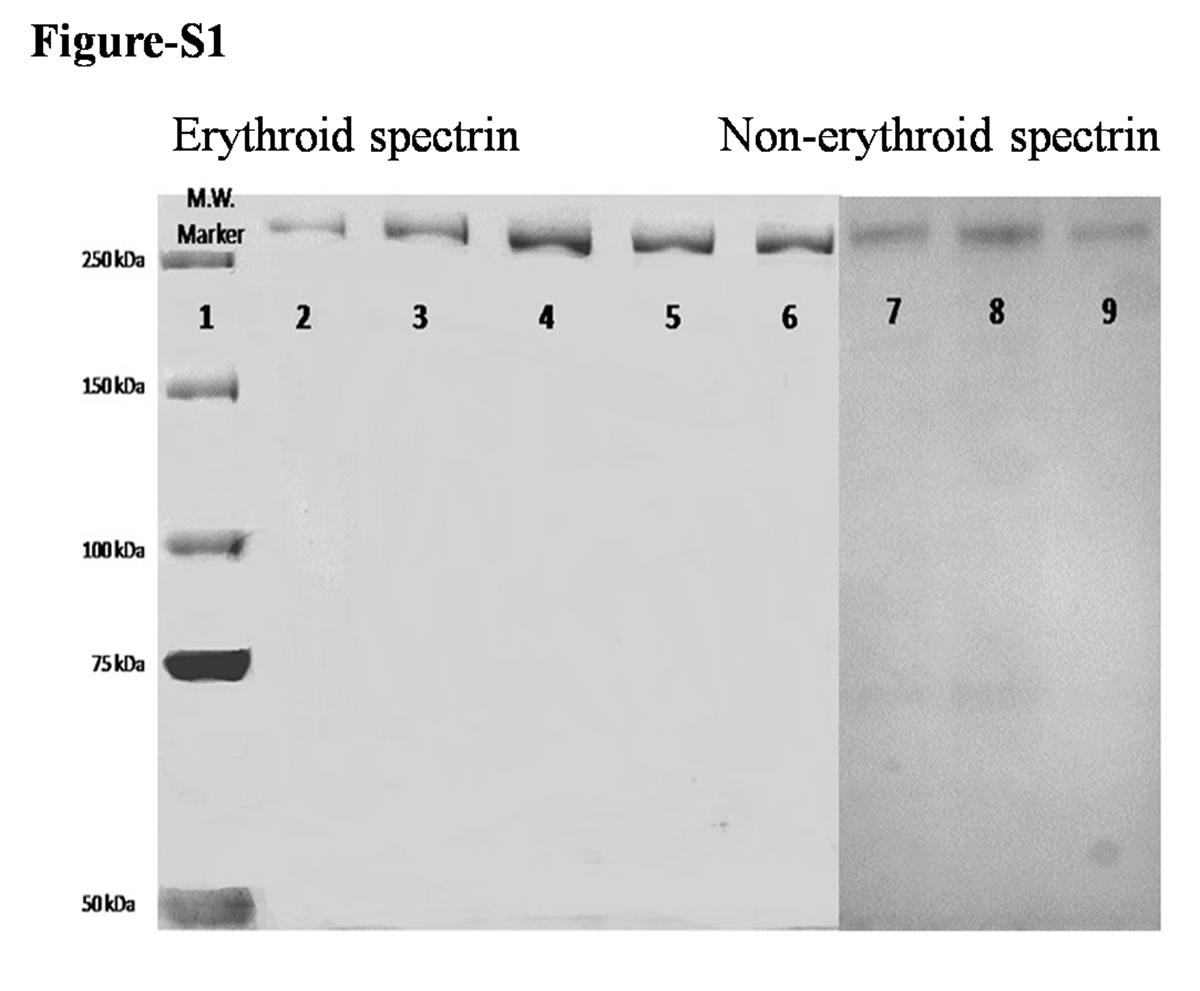

Supplement: S1 Fig — Lane (1) represent Molecular weight marker, lane (2) represent the protein after 1 hour incubation at 4°C, lane (3) at 25°C, lane 4, 5, and 6 represent erythroid spectrin after 1 hour incubation with 1M, 4M and 6M urea at 25°C. Lane 7, 8, and 9 represent the same for non-erythroid spectrin after 1 hour incubation with 1M, 4M and 6M urea at 25°C. (TIF) [file pone.0116991.s001.tif]

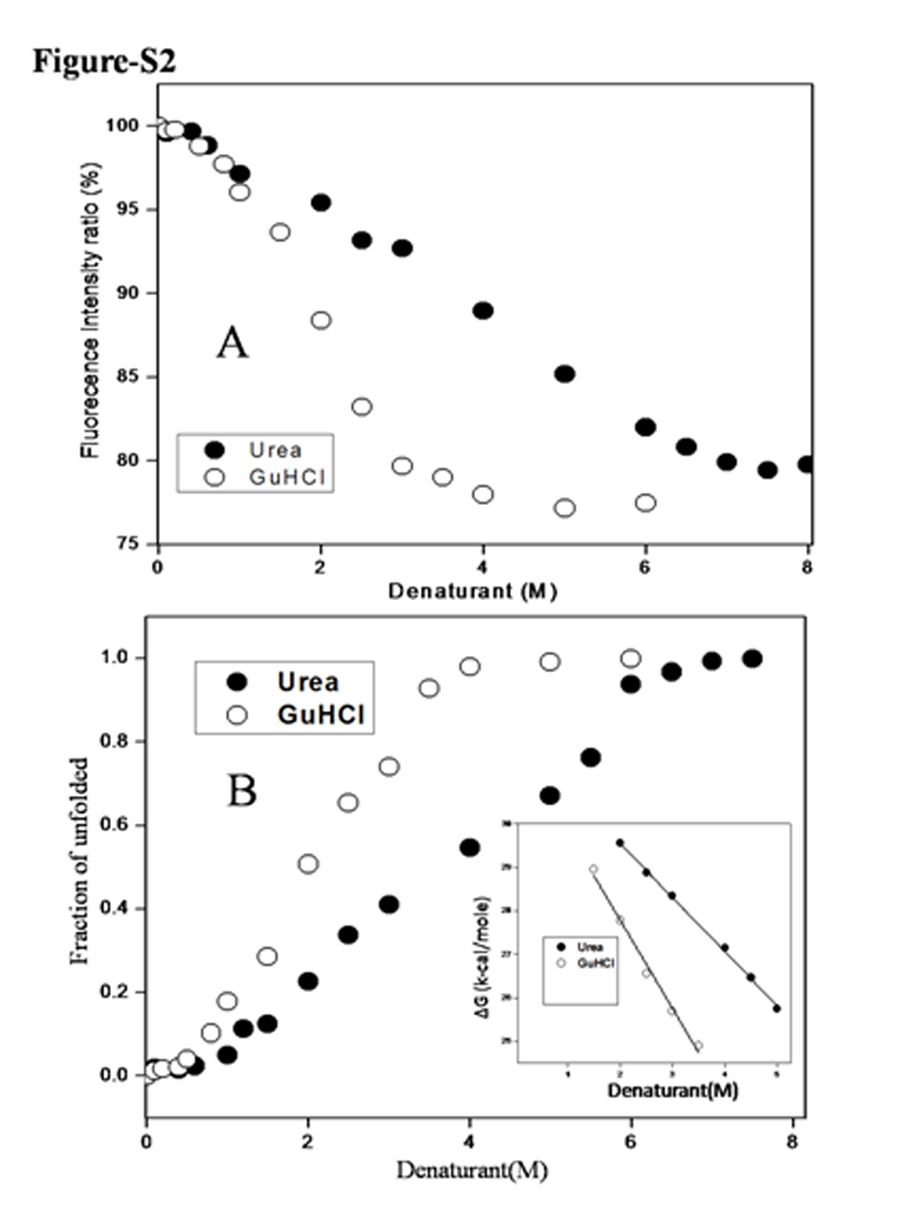

Supplement: S2 Fig — The data are represented as (A) the percentage of fluorescence taking the same for the native protein as 100% and (B) for the fraction of unfolded spectrin. Inset shows the linear free energy extrapolation curve with respect to increasing concentration of the denaturants. The ΔGD H 2 0 was obtained from the intercept on Y-axis by using linear extrapolation. (TIF) [file pone.0116991.s002.tif]

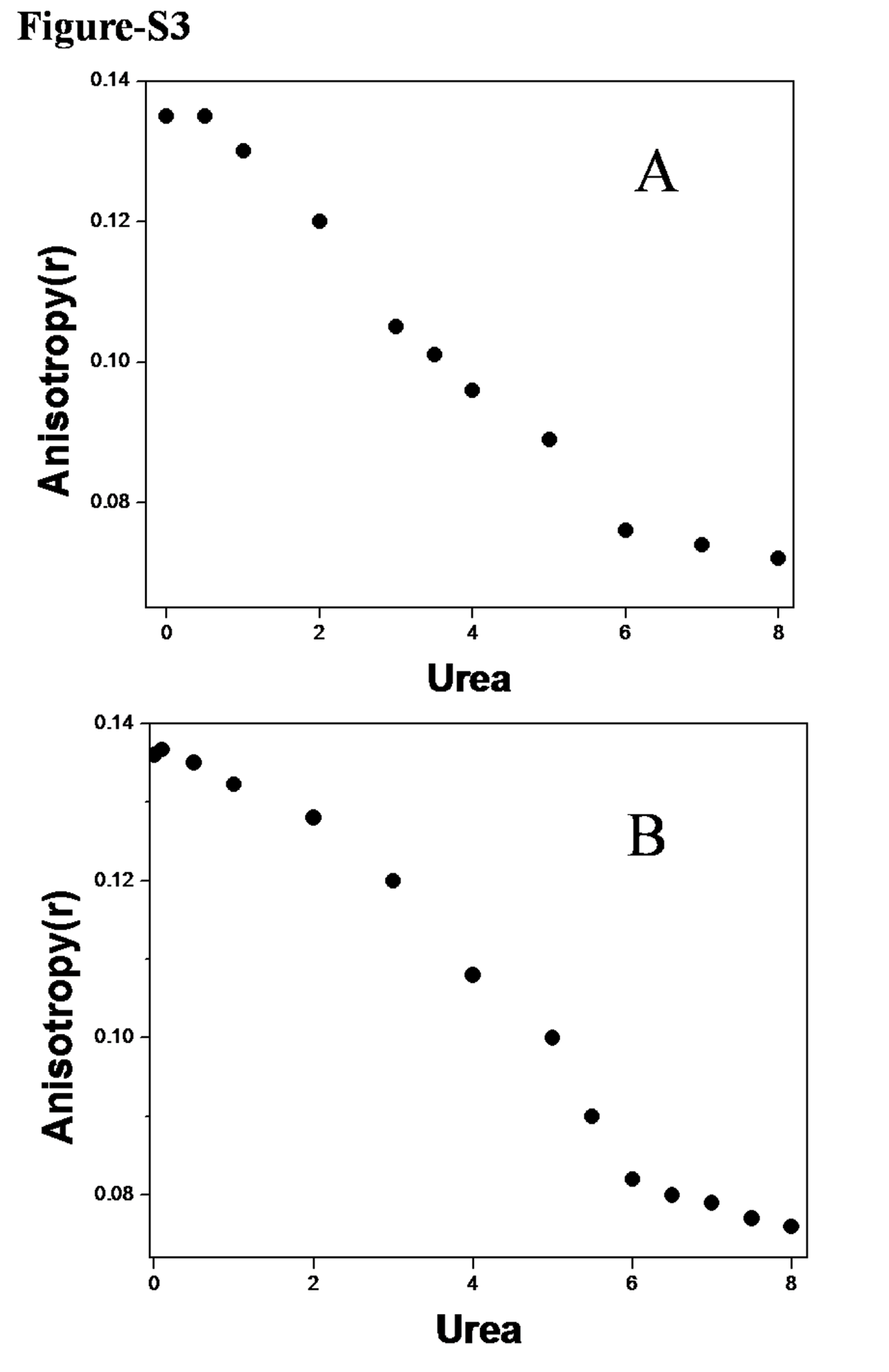

Supplement: S3 Fig — (TIF) [file pone.0116991.s003.tif]

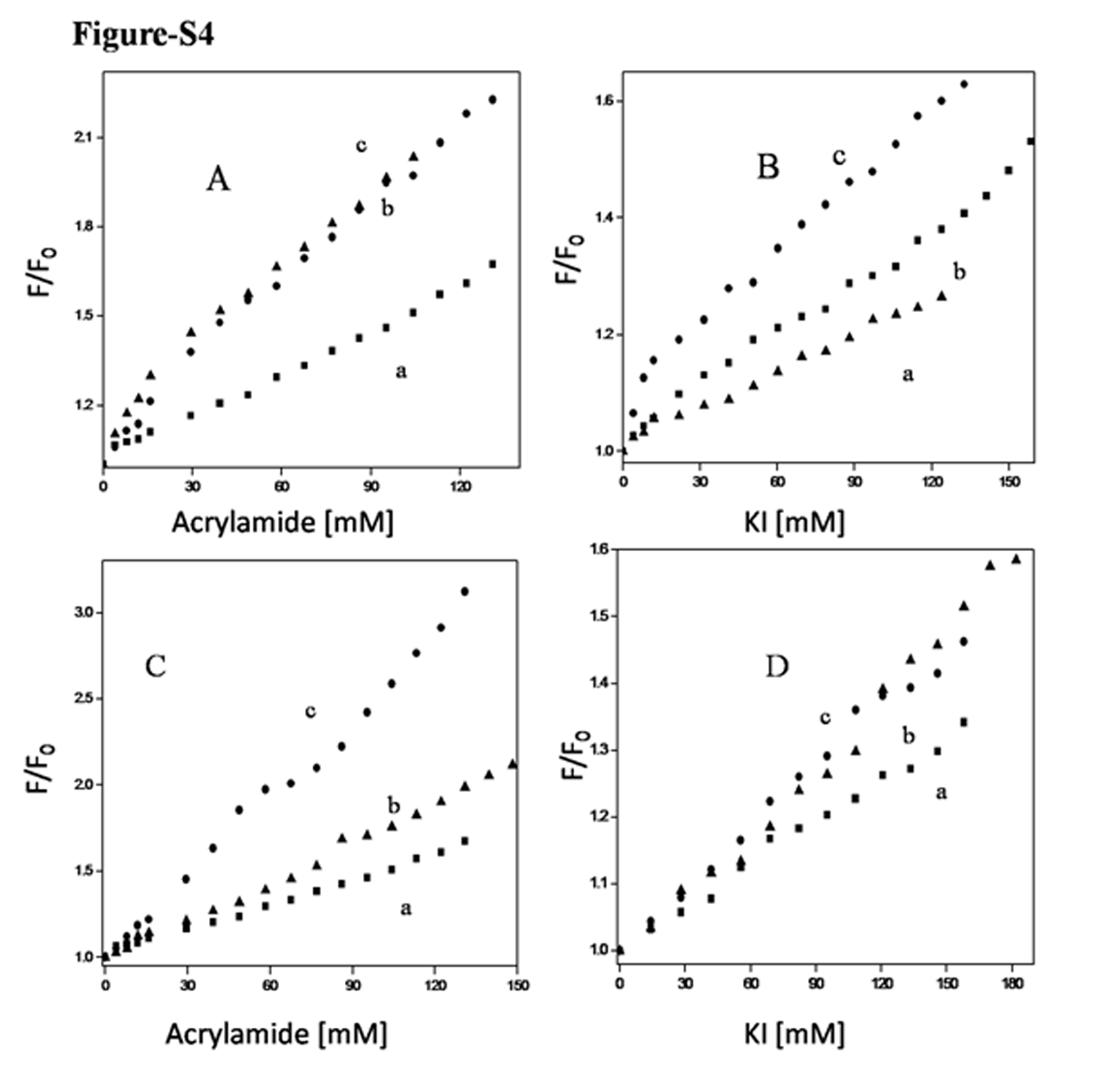

Supplement: S4 Fig — The data points (a) for native; (b) for denatured spectrin in 8M urea and (c) in 6M GuHCl. (TIF) [file pone.0116991.s004.tif]

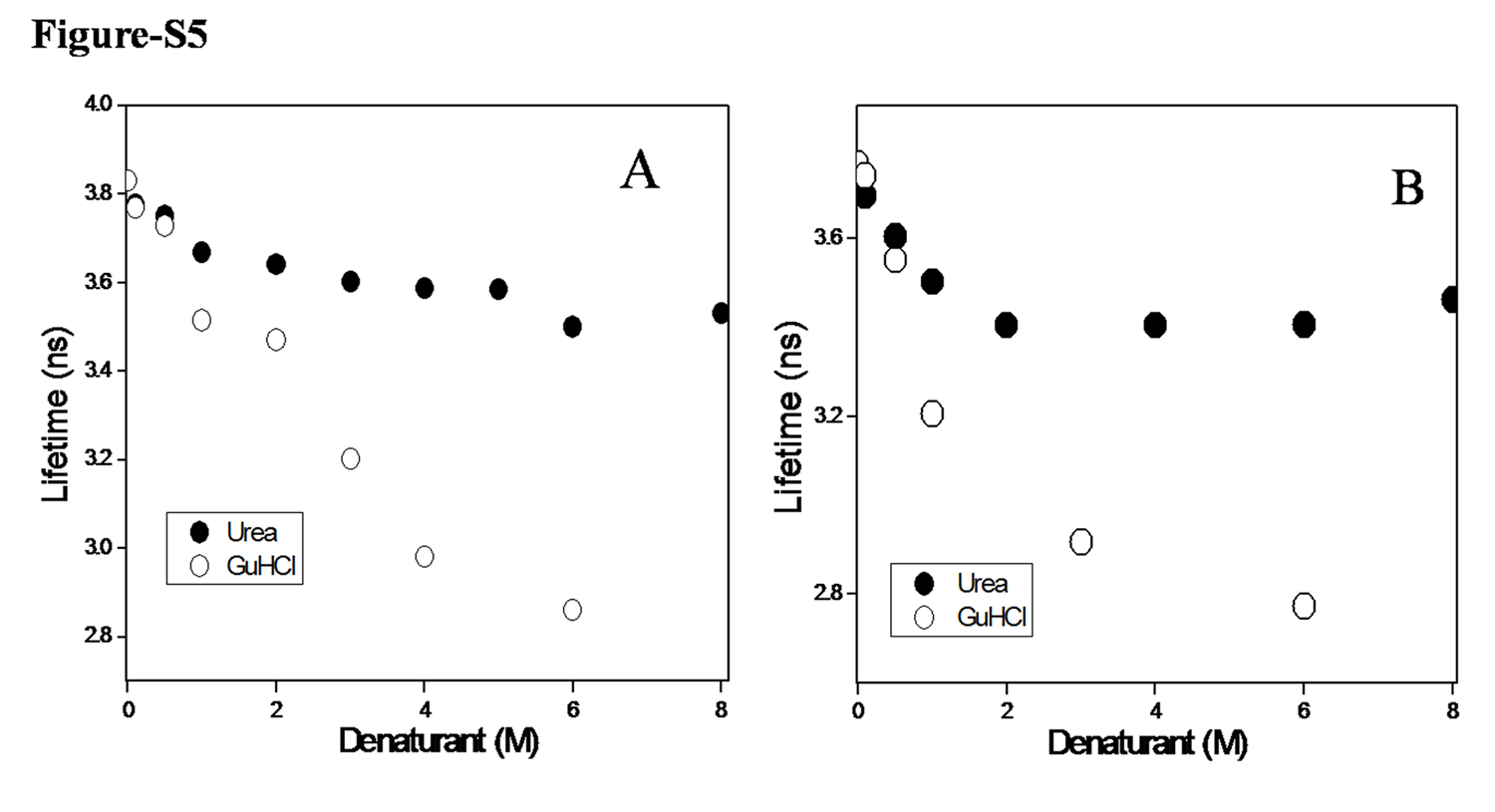

Supplement: S5 Fig — (TIF) [file pone.0116991.s005.tif]

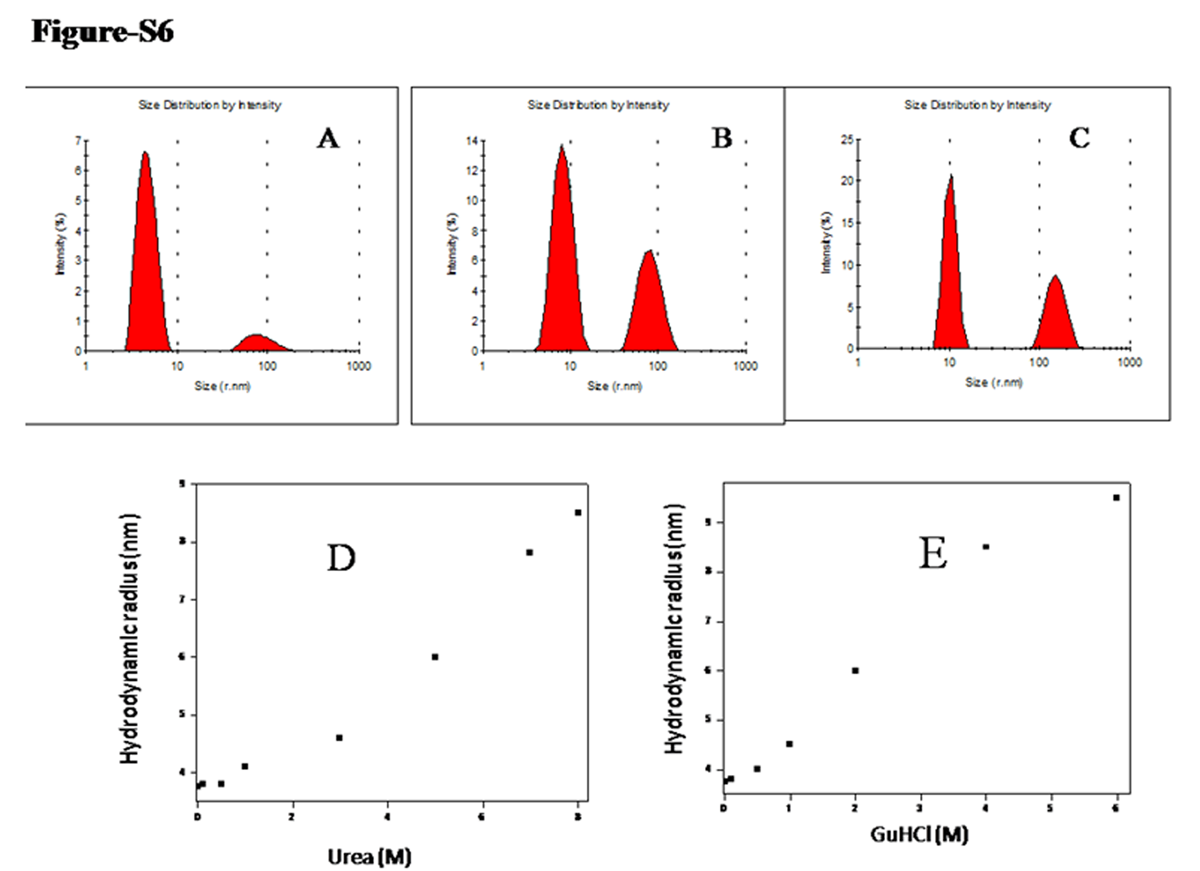

Supplement: S6 Fig — The figure in the bottom panel represents hydrodynamic radii of BSA with different concentration of (D) Urea (E) GuHCl. (TIF) [file pone.0116991.s006.tif]

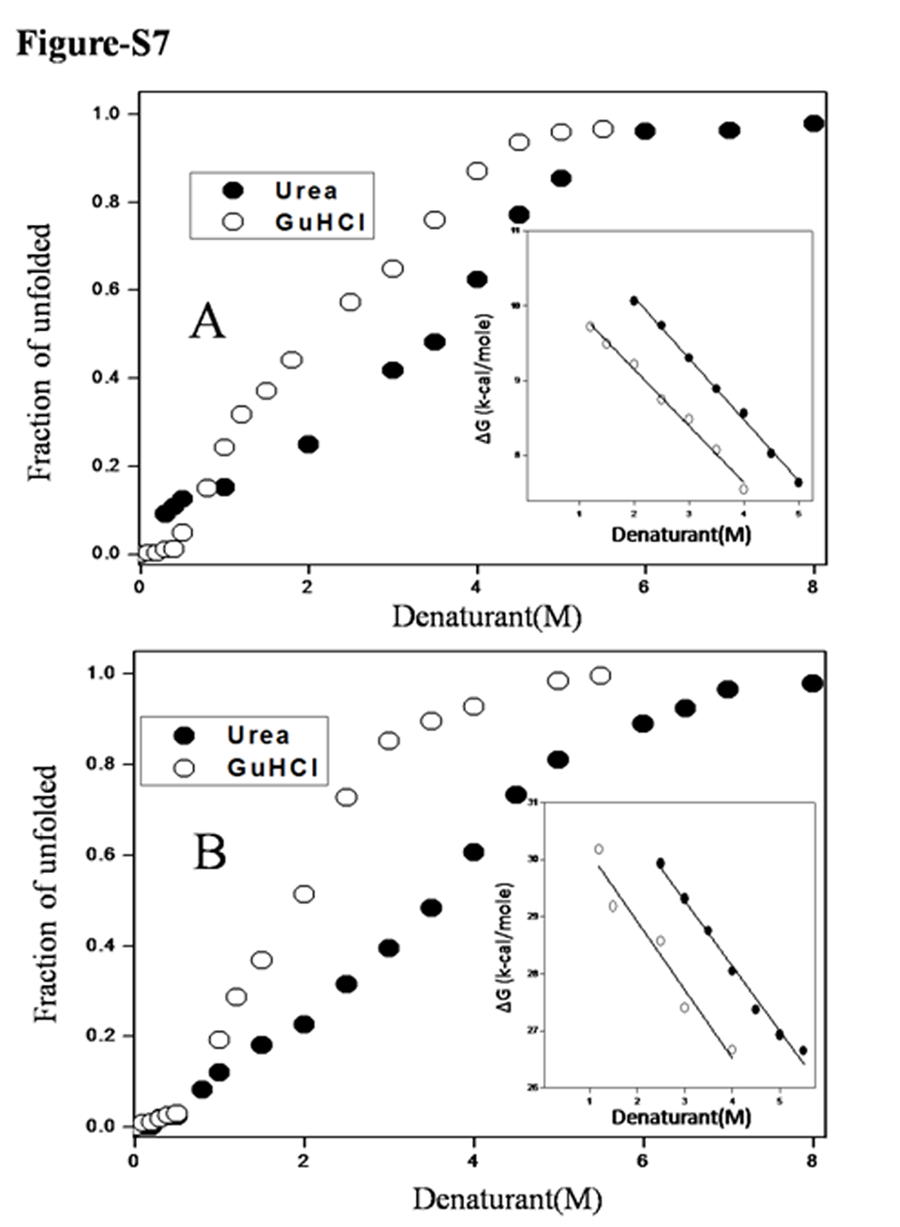

Supplement: S7 Fig — Insets show the linear free energy extrapolation curve with respect to increasing concentration of the denaturant concentrations. The ΔGD H20 was obtained from the intercept on Y-axis by using linear extrapolation. (TIF) [file pone.0116991.s007.tif]

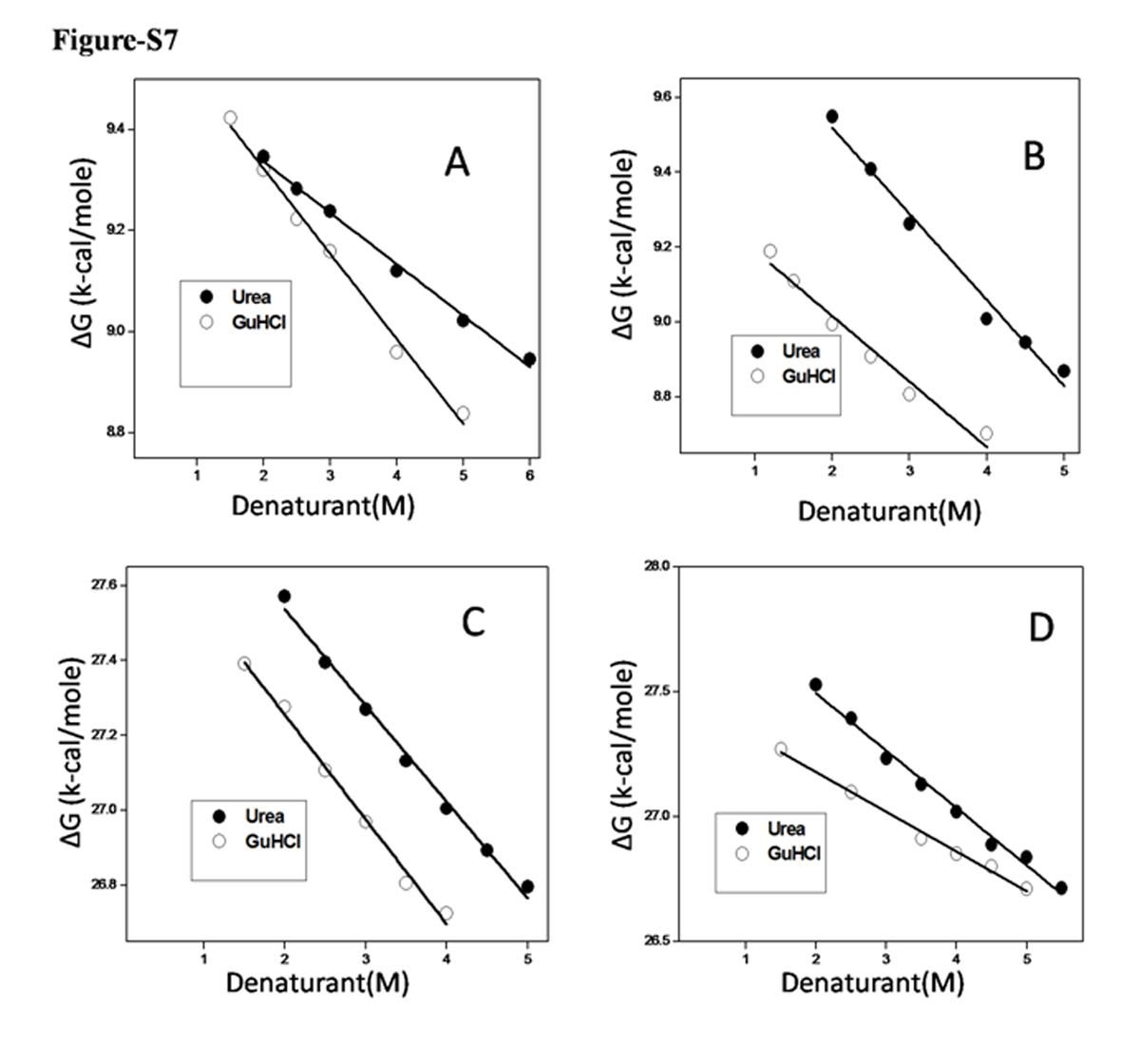

Supplement: S8 Fig — (TIF) [file pone.0116991.s008.tif]
