# Supplementary material for: Probing Conformational Stability and Dynamics of Erythroid and Nonerythroid Spectrin: Effects of Urea and Guanidine Hydrochloride
Source: PLoS One. 2015 Jan 24;10(1):e0116991. doi: 10.1371/journal.pone.0116991 (PMC4305312; doi:10.1371/journal.pone.0116991)
Supplement: S1 Table — KSV, fe, and kq in the presence and absence of the denaturants. (DOCX) [file pone.0116991.s009.docx]

Table-S1: The acrylamide quenching parameters of the tryptophan’s of erythroid, non-erythroid spectrin. K_SV_, f_e_, and k_q_ in the presence and absence of the denaturants.

| **Protein** | **Urea**  **(M)** | **K_SV_ (M^-1^S^-1^)** | **k_q_(M^.^ nS)^-1^** | **f_e_**  **(%)** | **GuHCl**  **(M)** | **K_SV_ (M^-1^S^-1^)** | **k_q_(M^.^ nS)^-1^** | **f_e_**  **(%)** |
| --- | --- | --- | --- | --- | --- | --- | --- | --- |
| Erythroid spectrin | 0  8 | 4.6±0.2  8.7±0.4 | 2.1±0.2  4.5±0.4 | 64  100 | 0  6 | 4.6±0.2  7.4±0.3 | 2.1±0.2  4.2±0.3 | 64  100 |
| Non-erythroid spectrin | 0  8 | 4.4±0.2  10.2±0.4 | 2.0±0.2  5.3±0.3 | 63  98 | 0  6 | 4.4±0.2  8.0±0.4 | 2.0±0.2  5.0±0.2 | 63  100 |
