# Supplementary material for: Probing Conformational Stability and Dynamics of Erythroid and Nonerythroid Spectrin: Effects of Urea and Guanidine Hydrochloride
Source: PLoS One. 2015 Jan 24;10(1):e0116991. doi: 10.1371/journal.pone.0116991 (PMC4305312; doi:10.1371/journal.pone.0116991)
Supplement: S2 Table — (DOCX) [file pone.0116991.s010.docx]

Table-S2: The iodide quenching parameters of the tryptophan’s of erythroid, non-erythroid spectrin tryptophan^’^s K_SV_ and K_q_ in the presence and absence of denaturant.

| **Protein** | **Urea**  **(M)** | **K_SV_ (M^-1^S^-1^)** | **k_q_(M^.^ nS)^-1^** | **GuHCl**  **(M)** | **K_SV_ (M^-1^S^-1^)** | **k_q_(M^.^ nS)^-1^** |
| --- | --- | --- | --- | --- | --- | --- |
| Erythroid spectrin | 0  8 | 2.2±0.2  3.2±0.2 | 1.5±0.2  2.6±0.4 | 0  6 | 2.2±0.2  4.01±0.3 | 1.5±0.2  3.4±0.3 |
| Non-erythroid spectrin | 0  8 | 1.7±0.2  2.98±0.4 | 1.6±0.2  2.4±0.3 | 0  6 | 1.7±0.2  3.40±0.4 | 1.6±0.2  3.33±0.2 |
