# Supplementary material for: Probing Conformational Stability and Dynamics of Erythroid and Nonerythroid Spectrin: Effects of Urea and Guanidine Hydrochloride
Source: PLoS One. 2015 Jan 24;10(1):e0116991. doi: 10.1371/journal.pone.0116991 (PMC4305312; doi:10.1371/journal.pone.0116991)
Supplement: S3 Table — (DOCX) [file pone.0116991.s011.docx]

Table-S3: Thermodynamic parameters of urea and GuHCl induced unfolding of erythroid and non-erythroid spectrin by direct fitting of the raw data.

| **Condition** | **Protein** | **Probe** | **ΔG^u^_H2O_**  **(Kcal/mole)** | **m _U-N_**  **(Kcal mole^-1^ M^-1^)** |
| --- | --- | --- | --- | --- |
| Urea | Erythroid spectrin  (Dimer)  Non-erythroid spectrin  (Tetramer) | Fluorescence  CD  Fluorescence  CD | 11.16  10.86  32.46  31.25 | -0.80  -0.78  -1.40  -1.24 |
| GuHCl | Erythroid spectrin  (Dimer)  Non-erythroid spectrin  (Tetramer) | Fluorescence  CD  Fluorescence  CD | 10.81  10.79  30.99  30.84 | -1.11  -1.06  -1.80  -1.25 |
